# Supplementary material for: Prevalence and risk factors of intestinal protozoal infections among patients in Malaysia: A systematic review and meta-analysis
Source: PLoS One. 2025 Sep 11;20(9):e0332218. doi: 10.1371/journal.pone.0332218 (PMC12425333; doi:10.1371/journal.pone.0332218)
Supplement: S1 Appendix — (DOCX) [file pone.0332218.s001.docx]

**S1 APPENDIX**

**Search strategy and sample search terms.**

A comprehensive literature search was carried out in the following databases from 2010 to 2024 conducted by the first author according (NSM) to Preferred Reporting Items for Systematic Reviews and Meta-Analyses (PRISMA) 2009 recommendations to locate studies in relevant databases, including PUBMED/MEDLINE, Scopus, Google Scholar, Web of Science, and Cochrane Library.

The results of each search were loaded into EndNote Volume X. (Clarivate Analytics, PA, USA). Medical Subject Headings and the keywords were used in the search machines were peer-reviewed between first author (NSM), senior supervising author (HAT) and another supervising author (WSM). Different keywords were chosen, and the search was conducted using ‘AND’ and ‘OR’ in the search section of the databases (Table 2). Reference lists from retrieved studies were used to identify more studies and were selected based on the systematic review inclusion criteria.

Studies were selected if they met the following criteria: Primary studies that were conducted in Malaysia, and reported on the type of diagnostic technique, risk factor, prevalence of intestinal protozoal infection, and type of clinical samples collected from patients. Studies were excluded if: Studies not conducted in Malaysia, samples specimens isolated from food, animals, and biotic components (water, air, light, soil and temperature), and the full text was not available. In addition, we also excluded case reports, reviews, or conference abstracts. Studies were selected based on the above inclusion and exclusion criteria by the main independent lead authors (S.M.). Disagreements were resolved by consensus between all authors. The details of the inclusion and exclusion criteria can be found in S1 Table.

The search results were yielded 49 population-based studies. Selection of studies was based on our stringent eligibility criteria, and it was verified by the senior supervising author (HAT). The evidence of robust search and studies selection was seen in a higher number of relevant studies, and no high-risk bias studies were included in our data.

**Research Question**

What is the overall prevalence of intestinal protozoan infection (IPI) and the risk factors among patients in Malaysia?

Table 1. PUBMED/MEDLINE search strategy.

| 1 | "Epidemiology"[Mesh] OR Prevalence*[tw] OR Incidence*[tw] OR Trend*[tw] OR Rate*[tw] OR "Epidemiological Stud*"[tw] OR "Cross-Sectional Stud*"[tw] OR "Observational Study*” [tw] |
| --- | --- |
| 2 | "Risk Factors"[Mesh] OR Risk factor*[tw] OR Mortalit*[tw] |
| 3 | "Protozoan Infections"[Mesh] OR "Intestinal Protozoa*"[tw] OR “Enteric protozoa*” [tw] OR Giardiasis[tw] OR Cryptosporidiosis[tw] OR Entamoebiasis[tw] OR Entamoeba[tw] OR Giardia[tw] OR Cryptosporidium[tw] |
| 4 | "Malaysia"[Mesh] OR “Peninsular Malaysia” [tw], “East Malaysia” [tw] |
| 5 | #1 AND/OR #2 AND #3 AND #4 |

Table 2. Cochrane Library search strategy.

| #1 | MeSH descriptor: [Epidemiology] explode all trees |
| --- | --- |
| #2 | ("prevalence"):ti,ab,kw OR ("incidence rate"):ti,ab,kw OR ("epidemiological studies"):ti,ab,kw |
| #3 | MeSH descriptor: [Risk Factors] explode all trees |
| #4 | ("risk factor"):ti,ab,kw OR ("mortality"):ti,ab,kw |
| #5 | MeSH descriptor: [Dysentery, Amebic] explode all trees |
| #6 | MeSH descriptor: [Giardiasis] explode all trees |
| #7 | MeSH descriptor: [Cryptosporidiosis] explode all trees |
| #8 | MeSH descriptor: [Malaysia] this term only |
| #9 | ("Malaysia"):ti,ab,kw OR ("Peninsular Malaysia"):ti,ab,kw OR ("East Malaysia"):ti,ab,kw |
| #10 | #1 OR #2 |
| #11 | #3 OR #4 |
| #12 | #5 OR #6 OR #7 |
| #13 | #8 OR #9 |
| #14 | #10 AND #5 |
| #15 | #10 AND #6 |
| #16 | #10 AND #7 |
| #17 | #14 OR #15 OR #16 |

Table 3. Scopus search database.

| 1 | Epidemiolog* or Prevalence* or Incidence* or Trend* or Rate* or "Epidemiological Stud*" or "Cross-Sectional Stud*" or "Observational Study*” |
| --- | --- |
| 2 | "Protozoan Infections" or "Intestinal Protozoa*" or “Enteric protozoa*” or Giardiasis or Cryptosporidiosis or Entamoebiasis or Entamoeba or Giardia or Cryptosporidium |
| 3 | Malaysia or “Peninsular Malaysia” or “East Malaysia” |
| 4 | ( TITLE-ABS-KEY ( epidemiolog* OR prevalence* OR incidence* OR trend* OR rate* OR "Epidemiological Stud*" OR "Cross-Sectional Stud*" OR "Observational Study*" ) AND TITLE-ABS-KEY ( "Protozoan Infections" OR "Intestinal Protozoa*" OR "Enteric protozoa*" OR giardiasis OR cryptosporidiosis OR entamoebiasis OR entamoeba OR giardia OR cryptosporidium ) AND TITLE-ABS-KEY ( malaysia OR "Peninsular Malaysia" OR "East Malaysia" ) ) AND PUBYEAR > 2010 AND PUBYEAR < 2025 |

Table 4 WOS search database.

| 1 | Epidemiolog* or Prevalence* or Incidence* or Trend* or Rate* or "Epidemiological Stud*" or "Cross-Sectional Stud*" or "Observational Study*” |
| --- | --- |
| 2 | "Protozoan Infections" or "Intestinal Protozoa*" or “Enteric protozoa*” or Giardiasis or Cryptosporidiosis or Entamoebiasis or Entamoeba or Giardia or Cryptosporidium |
| 3 | Malaysia or “Peninsular Malaysia” or “East Malaysia” |
| 4 | Epidemiolog* or Prevalence* or Incidence* or Trend* or Rate* or "Epidemiological Stud*" or "Cross-Sectional Stud*" or "Observational Study*” (Topic) and "Protozoan Infections" or "Intestinal Protozoa*" or “Enteric protozoa*” or Giardiasis or Cryptosporidiosis or entamebiasis or Entamoeba or Giardia or Cryptosporidium (All Fields) and Malaysia or “Peninsular Malaysia” or “East Malaysia” (All Fields) and Review Article (Exclude – Document Types) and 2024 or 2010 or 2016 or 2017 or 2018 or 2019 or 2020 or 2021 or 2022 or 2023 or 2011 or 2012 or 2013 or 2014 or 2015 (Publication Years) \| 119 results |
