# Supplementary material for: Prevalence and risk factors of intestinal protozoal infections among patients in Malaysia: A systematic review and meta-analysis
Source: PLoS One. 2025 Sep 11;20(9):e0332218. doi: 10.1371/journal.pone.0332218 (PMC12425333; doi:10.1371/journal.pone.0332218)
Supplement: S2 Appendix — (DOCX) [file pone.0332218.s002.docx]

**S2 APPENDIX**

**Included studies**

**Table 3. Chronological overview of the 49 published population-based studies that were included in the meta-analysis review.**

| No | Author | Year | Region | Detection Method | Total sample | Overall Prevalence | Study Population | Total Score | Quality Rating |
| --- | --- | --- | --- | --- | --- | --- | --- | --- | --- |
| 1 | Siti Farah Norasyikeen et al., | 2024 | Kuala Lumpur | Microscopy | 134 | 0.082 | Hospitalized patients | 0 | Low risk |
| 2 | Mohd Hanapi et al., | 2023 | Selangor | Microscopy | 418 | 0.077 | Migrants | 0 | Low risk |
| 3 | Zamari et al., | 2023 | Selangor | Microscopy | 37 | 0.676 | Local community | 3 | Low risk |
| 4 | Saidin et al., | 2022 | Perak | Molecular | 55 | 0.164 | Aboriginal people | 0 | Low risk |
| 5 | Mohamed Kamel et al., | 2022 | Perak | Microscopy | 99 | 0.091 | Aboriginal people | 4 | Moderate risk |
| 6 | Tokijoh et al., | 2022 | Perak, | Molecular | 544 | 0.213 | Aboriginal people | 0 | Low risk |
| 7 | Tokijoh et al.,b | 2021 | Perak | Molecular | 453 | 0.647 | Aboriginal people | 0 | Low risk |
| 8 | Sahimin et al., | 2020 | Peninsular Malaysia | Microscopy | 206 | 0.029 | Local communities | 0 | Low risk |
| 9 | Mohamed Nur Adli & Mohamed Kamel, | 2020 | Pahang | Microscopy | 208 | 0.346 | Aboriginal people | 4 | Moderate risk |
| 10 | Tang et al., | 2020 | Perak, | Microscopy | 116 | 0.681 | Aboriginal people | 4 | Moderate risk |
| 11 | Sahimin et al.,b | 2019 | Peninsular Malaysia | Microscopy, Immunoassay, Molecular | 306 | 0.386 | Migrants | 3 | Low risk |
| 12 | Jeyaprakasam et al., | 2019 | Perak | Microscopy | 139 | 0.151 | Aboriginal people | 4 | Moderate risk |
| 13 | Asady Abdullah et al., | 2019 | Pahang | Microscopy | 135 | 0.252 | Hospitalized patients | 0 | Low risk |
| 14 | Noradilah et al., | 2019 | Pahang | Microscopy | 473 | 0.104 | Aboriginal people | 0 | Low risk |
| 15 | Mohamed Kamel et al.,b | 2018 | Pahang | Microscopy | 111 | 0.234 | Aboriginal people | 4 | Moderate risk |
| 16 | Hartini & Mohamed Kamel., | 2018 | Kuala Lumpur | Microscopy | 171 | 0.234 | Hospitalized patients | 4 | Moderate risk |
| 17 | Rajoo et al., | 2017 | Sarawak | Microscopy | 341 | 0.103 | Aboriginal people | 0 | Low risk |
| 18 | Saidin et al., | 2017 | Kelantan | Microscopy, Immunoassay, Molecular | 70 | 0.657 | Hospitalized patients | 0 | Low risk |
| 19 | Sahimin et al., c | 2016 | Peninsular Malaysia | Microscopy | 388 | 0.255 | Migrants | 0 | Low risk |
| 20 | Elyana et al., | 2016 | Terengganu | Microscopy | 340 | 0.259 | Aboriginal people | 0 | Low risk |
| 21 | Chin et al., | 2016 | Selangor | Microscopy, Molecular | 186 | 0.172 | Aboriginal people | 0 | Low risk |
| 22 | Anuar et al., | 2016 | Pahang | Microscopy | 255 | 0.149 | Aboriginal people | 4 | Moderate risk |
| 23 | Mohamed Kamel et al., c | 2016 | Kelantan | Microscopy | 111 | 0.342 | Aboriginal people | 4 | Moderate risk |
| 24 | Wong et al., | 2016 | Peninsular Malaysia | Immunoassay | 375 | 0.709 | Aboriginal people | 0 | Low risk |
| 25 | Nisha et al., | 2015 | Selangor | Microscopy | 110 | 0.073 | Aboriginal people | 4 | Moderate risk |
| 26 | Angal et al., | 2015 | Selangor | Microscopy | 294 | 0.054 | Migrants | 0 | Low risk |
| 27 | Asma et al., | 2015 | Peninsular Malaysia | Microscopy, Molecular | 346 | 0.124 | Hospitalized patients | 0 | Low risk |
| 28 | Ahmed Al-Delaimy et al., | 2014 | Pahang | Microscopy | 498 | 0.476 | Aboriginal people | 0 | Low risk |
| 29 | Lee et al., | 2014 | Peninsular Malaysia | Microscopy | 269 | 0.182 | Aboriginal people | 0 | Low risk |
| 30 | Ahmad et al., | 2014 | Perak | Microscopy | 131 | 0.008 | Local communities | 5 | Moderate risk |
| 31 | Choy et al., | 2014 | Peninsular Malaysia | Microscopy | 1330 | 0.281 | Aboriginal people | 0 | Low risk |
| 32 | Anuar et al., b | 2014 | Peninsular Malaysia | Microscopy, Molecular | 611 | 0.16 | Aboriginal people | 0 | Low risk |
| 33 | Hanapian et al., | 2014 | Perak | Microscopy | 175 | 0.594 | Aboriginal people | 4 | Moderate risk |
| 34 | Anuar et al., c | 2013 | Peninsular Malaysia | Microscopy | 447 | 0.058 | Aboriginal people | 0 | Low risk |
| 35 | Hartini et al., | 2013 | Kelantan | Microscopy | 111 | 0.45 | Aboriginal people | 4 | Moderate risk |
| 36 | Lau et al., | 2013 | Peninsular Malaysia | Molecular | 334 | 0.195 | Aboriginal people | 0 | Low risk |
| 37 | Al-Mekhlafi et al., | 2013 | Pahang | Microscopy | 374 | 0.313 | Local communities | 0 | Low risk |
| 38 | Al-Harazi et al., | 2013 | Pahang | Microscopy | 307 | 0.365 | Aboriginal people | 4 | Moderate risk |
| 39 | Basuni et al., | 2012 | Kelantan | Microscopy, Molecular | 225 | 0.08 | Hospitalized patients | 0 | Low risk |
| 40 | Sinniah et al., | 2012 | Perak | Microscopy | 77 | 0.078 | Aboriginal people | 4 | Moderate risk |
| 41 | Ngui et al., | 2012 | Peninsular Malaysia | Microscopy, Molecular | 426 | 0.176 | Local communities | 0 | Low risk |
| 42 | Rossle et al., | 2012 | Selangor | Microscopy, Immunoassay | 130 | 0.046 | Hospitalized patients | 4 | Moderate risk |
| 43 | Anuar et al., d | 2012 | Peninsular Malaysia | Microscopy | 500 | 0.186 | Aboriginal people | 0 | Low risk |
| 44 | Asma et al., b | 2011 | Peninsular Malaysia | Microscopy | 346 | 0.324 | Hospitalized patients | 2 | Low risk |
| 45 | Ngui et al., b | 2011 | Peninsular Malaysia | Microscopy | 716 | 0.228 | Local communities | 0 | Low risk |
| 46 | Lim et al., | 2011 | Selangor | Microscopy, Molecular | 122 | 0.279 | Hospitalized patients | 0 | Low risk |
| 47 | Lono et al., | 2011 | Selangor | Microscopy, Molecular | 247 | 0.04 | Hospitalized patients | 0 | Low risk |
| 48 | Al-Mekhlafi et al., b | 2011 | Selangor | Microscopy | 276 | 0.072 | Aboriginal people | 4 | Medium risk |
| 49 | Al-Mekhlafi et al., c | 2010 | Pahang | Microscopy | 241 | 0.216 | Aboriginal people | 4 | Medium risk |

**References**

(1-49)

1. Siti Farah Norasyikeen SO, Ngui R, Syaza Zafirah AR, Md Zoqratt MZH, Eng WWH, Ayub Q, et al. Study on intestinal parasitic infections and gut microbiota in cancer patients at a tertiary teaching hospital in Malaysia. Sci Rep. 2024;14(1):13650.
2. Mohd Hanapi IR, Behnke JM, Sahimin N, Saifulazmi NF, Golam Mohammad Khan ASJ, Abdul Mutalib RNS, et al. Intestinal parasitic infections and risk analysis among urban refugees in the Klang Valley, Malaysia. Trans R Soc Trop Med Hyg. 2024;118(1):18-32.
3. Zamari NZA, Rosli N, Nadiah N, Pin TM, Zain SNM, Davamani F, Nisha M. First report on gastrointestinal parasite (GIP) infection among geriatric community with cognitive impairment in Selangor, Malaysia. Malaysian Journal of Public Health Medicine. 2023;23(1):146-52.
4. Tokijoh NI, Bakar AA, Othman N, Noordin R, Saidin S. Assessing the prevalence and risk factors associated with Entamoeba complex infection among the Orang Asli school children in Perak, Malaysia through molecular approach. Parasitol Int. 2022;91:102638.
5. Tokijoh NI, Saidin S, Abu Bakar A, Othman N, Noordin R. PCR detection of entamoeba using genus-specific primers from Orang Asli schoolchildren in Perak. Bioscience Research. 2021;18:295-307.
6. Saidin S, Adibah AB, Poh KB, Khairuddin R, Md-Zain BM. Molecular prevalence of *Entamoeba histolytica* and *Entamoeba dispa*r infection among aboriginal communities in the southern region of Perak, Malaysia. IIUM Medical Journal Malaysia. 2022;21(1):30-8.
7. Mohamed Kamel AG, Ying LS, Tien NK, Anne Y, Peng TS. Amoebiasis amongst the Orang Asli (Aborigine) schoolchildren of Pos Kuala Mu, Sungai Siput, Perak, Malaysia. Buletin Sains Kesihatan. 2022;6(2):19-23.
8. Tang SGH, Mohamed Kamel AG. Intestinal protozoan infections of schoolchildren in an Aboriginal (Orang Asli) settlement in Perak, Malaysia. International Medical Journal. 2020;27(1):31-5.
9. Sahimin N, Abd Khalil NS, Lewis JW, Mohd Zain SN. Post-era mass drug administration: An update on intestinal parasitic infections in urban poor communities in Peninsular Malaysia. Tropical Biomedicine. 2020;37(2):363-71.
10. Adli MN, Ghani MKA. Giardiasis amongst the orang asli (Aborigine) school children at pos senderut, Pahang, Malaysia. International Medical Journal. 2020;27(1):28-30.
11. Sahimin N, Yunus MH, Douadi B, Lim ALY, Noordin R, Behnke JM, Zain SNM. Entamoeba infections and associated risk factors among migrant workers in Peninsular Malaysia. Tropical Biomedicine. 2019;36(4):1014-26.
12. Noradilah SA, Moktar N, Lee IL, Salleh FM, Anuar TS. Impact of seasonality on the prevalence and risk factors of Giardia lamblia infections among the aborigines. Asian Pacific Journal Of Tropical Medicine. 2019;12(7):315-20.
13. Jeyaprakasam NK, Ghani MKA. Giardiasis amongst the aboriginal school children at Sungai Raba village Gerik, Perak, Malaysia. International Medical Journal. 2019;26(5):399-401.
14. Asady A, Ismail S, Marsitah AJ, Pakeer O. Prevalence of *Cryptosporidium spp.* infection among children admitted to Hospital Tengku Ampuan Afzan. Med J Malaysia. 2019;74(6):468-71.
15. Yusof H, Mohamed Kamel AG. Parasitic Infections Among Renal Transplant Recipients in Kuala Lumpur Hospital. Jurnal Sains Kesihatan Malaysia. 2018;16(1):57-61.
16. Mohamed Kamel AG, Musa A. Giardiasis amongst the orang asli School Children at Pos Titom, Pahang, Malaysia. Jurnal Sains Kesihatan Malaysia. 2018:89-93.
17. Saidin S, Yunus MH, Othman N, Lim YAL, Mohamed Z, Zakaria NZ, Noordin R. Development and initial evaluation of a lateral flow dipstick test for antigen detection of *Entamoeba histolytica* in stool sample. Pathogens and Global Health. 2017;111(3):128-36.
18. Rajoo Y, Ambu S, Lim YAL, Rajoo K, Tey SC, Lu CW, Ngui R. Neglected intestinal parasites, malnutrition and associated key factors: A population based cross-sectional study among indigenous communities in sarawak, Malaysia. Plos One. 2017;12(1).
19. Wong WK, Lim BH, Jan JMH, Foo PC, Dony JF, Zeehaida M, Noordin R. Prevalence and distribution of anti-amoebic IgG antibody among Orang Asli (Aborigines) in Peninsular Malaysia. Tropical Biomedicine. 2016;33(4):739-45.
20. Sahimin N, Lim YAL, Ariffin F, Behnke JM, Lewis JW, Zain SNM. Migrant workers in malaysia: current implications of sociodemographic and environmental characteristics in the transmission of intestinal parasitic infections. Plos Neglected Tropical Diseases. 2016;10(11).
21. Mohamed Kamel AG, Gopal G, Zawawi MA, Yusof H, Haron N. High prevalence of cryptosporidiosis amongst the Orang Asli (aborigine) children at Sungai Rual Post, Kelantan, Malaysia. International Medical Journal. 2016;23:553-5.
22. Elyana FN, Al-Mekhlafi HM, Ithoi I, Abdulsalam AM, Dawaki S, Nasr NA, et al. A tale of two communities: intestinal polyparasitism among Orang Asli and Malay communities in rural Terengganu, Malaysia. Parasites and Vectors. 2016;9(1).
23. Chin YT, Lim YAL, Chong CW, Teh CSJ, Yap IKS, Lee SC, et al. Prevalence and risk factors of intestinal parasitism among two indigenous sub-ethnic groups in Peninsular Malaysia. Infectious Diseases Of Poverty. 2016;5.
24. Anuar TS, Bakar NHA, Al-Mekhlafi HM, Moktar N, Osman E. Prevalence and risk factors for asymptomatic intestinal microsporidiosis among aboriginal school children in Pahang, Malaysia. Southeast Asian Journal of Tropical Medicine and Public Health. 2016;47(3):441-9.
25. Nisha M, Kumarasamy V, Ambu S, Davamani F, Mak JW. Factors Associated with Intestinal Parasite Infections in a Resettled Indigenous Community in Malaysia. International Journal of Tropical Diseases & Health. 2015;12(3).
26. Asma I, Sim BL, Brent RD, Johari S, Yvonne Lim AL. Molecular epidemiology of Cryptosporidium in HIV/AIDS patients in Malaysia. Trop Biomed. 2015;32(2):310-22.
27. Angal L, Mahmud R, Samin S, Yap NJ, Ngui R, Amir A, et al. Determining intestinal parasitic infections (IPIs) in inmates from Kajang Prison, Selangor, Malaysia for improved prison management. BMC Infectious Diseases. 2015;15(1).
28. Lee SC, Ngui R, Tan TK, Aidil RM, Lim YAL. Neglected tropical diseases among two indigenous subtribes in Peninsular Malaysia: highlighting differences and co-infection of helminthiasis and sarcocystosis. Plos One. 2014;9(9).
29. Hanapian YL, Mak JW, Chen PCY. An intestinal parasitological survey among the Jehai Orang Asli (aborigines) of theTemenggor forest, Perak state, Malaysia. International e-Journal of Science, Medicine and Education (IeJSME). 2014;18.
30. Choy SH, Al-Mekhlafi HM, Mahdy MAK, Nasr NN, Sulaiman M, Lim YAL, Surin J. Prevalence and associated risk factors of giardia infection among indigenous communities in rural Malaysia. Scientific Reports. 2014;4.
31. Anuar TS, Azreen SN, Salleh FM, Moktar N. Molecular epidemiology of giardiasis among Orang Asli in Malaysia: application of the triosephosphate isomerase gene. BMC Infectious Diseases. 2014;14.
32. Al-Delaimy AK, Al-Mekhlafi HM, Nasr NA, Sady H, Atroosh WM, Nashiry M, et al. Epidemiology of intestinal polyparasitism among Orang Asli school children in rural Malaysia. PLoS Neglected Tropical Diseases. 2014;8(8).
33. Ahmad AF, Ngui R, Muhammad Aidil R, Lim YA, Rohela M. Current status of parasitic infections among Pangkor Island community in Peninsular Malaysia. Tropical biomedicine. 2014;31(4):836-43.
34. Lau YL, Anthony C, Fakhrurrazi SA, Ibrahim J, Ithoi I, Mahmud R. Real-time PCR assay in differentiating *Entamoeba histolytica, Entamoeba dispar*, and *Entamoeba moshkovskii* infections in Orang Asli settlements in Malaysia. Parasites & Vectors. 2013;6.
35. Hartini Y, Geishamimi G, Mariam AZ, Mohamed-Kamel AG, Hidayatul FO, Ismarul YI. Distribution of intestinal parasitic infections amongst aborigine children at Post Sungai Rual, Kelantan, Malaysia. Tropical Biomedicine. 2013;30(4):596-601.
36. Anuar TS, Al-Mekhlafi HM, Salleh FM, Moktar N. New Insights of Microsporidial Infection among Asymptomatic Aboriginal Population in Malaysia. PLOS One. 2013;8(8).
37. Al-Mekhlafi HM, Al-Maktari MT, Jani R, Ahmed A, Anuar TS, Moktar N, et al. Burden of *Giardia duodenalis* Infection and its adverse effects on growth of schoolchildren in rural Malaysia. PLOS Neglected Tropical Diseases. 2013;7(10).
38. Al-Harazi T, Ghani MK, Othman H. Prevalence of intestinal protozoan infections among Orang Asli schoolchildren in Pos Senderut, Pahang, Malaysia. Journal of the Egyptian Society of Parasitology. 2013;43(3):561-8.
39. Sinniah B, Sabaridah I, Soe MM, Sabitha P, Awang IPR, Ong GP, Hassan AKR. Determining the prevalence of intestinal parasites in three Orang Asli (Aborigines) communities in Perak, Malaysia. Tropical Biomedicine. 2012;29(2):200-6.
40. Rossle NF, Latif B, Malik MA, Abu Bakar A. Cryptosporidiosis among children with diarrhea admitted to Hospital Selayang and Hospital Sungai Buloh, Selangor, Malaysia. J Trop Med Parasitol. 2012;35:55-62.
41. Ngui R, Angal L, Fakhrurrazi SA, Lian YLA, Ling LY, Ibrahim J, Mahmud R. Differentiating *Entamoeba histolytica, Entamoeba dispar* and *Entamoeba moshkovskii* using nested polymerase chain reaction (PCR) in rural communities in Malaysia. Parasites & Vectors. 2012;5.
42. Basuni M, Mohamed Z, Ahmad M, Zakaria NZ, Noordin R. Detection of selected intestinal helminths and protozoa at Hospital Universiti Sains Malaysia using multiplex real-time PCR. Tropical Biomedicine. 2012;29(3):434-42.
43. Anuar TS, Al-Mekhlafi HM, Abdul Ghani MK, Abu Bakar E, Azreen SN, Salleh FM, et al. Molecular epidemiology of amoebiasis in Malaysia: highlighting the different risk factors of *Entamoeba histolytica* and *Entamoeba dispar* infections among Orang Asli communities. Int J Parasitol. 2012;42(13-14):1165-75.
44. Ngui R, Ishak S, Chuen CS, Mahmud R, Lim YAL. Prevalence and risk factors of intestinal parasitism in rural and remote West Malaysia. Plos Neglected Tropical Diseases. 2011;5(3).
45. Lono A, Kumar S, Chye TT. Detection of microsporidia in local HIV-positive population in Malaysia. Transactions Of The Royal Society Of Tropical Medicine And Hygiene. 2011;105(7):409-13.
46. Lim YAL, Iqbal A, Surin J, Sim BLH, Jex AR, Nolan MJ, et al. First genetic classification of Cryptosporidium and Giardia from HIV/AIDS patients in Malaysia. Infection Genetics And Evolution. 2011;11(5):968-74.
47. Asma I, Johari S, Sim BL, Lim YA. How common is intestinal parasitism in HIV-infected patients in Malaysia? Trop Biomed. 2011;28(2):400-10.
48. Al-Mekhlafi HM, Mahdy MA, Azlin MY, Fatmah MS, Norhayati M. Childhood Cryptosporidium infection among aboriginal communities in Peninsular Malaysia. Ann Trop Med Parasitol. 2011;105(2):135-43.
49. Al-Mekhlafi HM, Sunn J, Sallam AA, Abdullah AW, Mahdy MAK. Giardiasis and poor vitamin a status among aboriginal school children in rural Malaysia. American Journal Of Tropical Medicine And Hygiene. 2010;83(3):523-7.
