# Supplementary material for: Prevalence and risk factors of intestinal protozoal infections among patients in Malaysia: A systematic review and meta-analysis
Source: PLoS One. 2025 Sep 11;20(9):e0332218. doi: 10.1371/journal.pone.0332218 (PMC12425333; doi:10.1371/journal.pone.0332218)
Supplement: S3 Appendix — (DOCX) [file pone.0332218.s003.docx]

**S3 APPENDIX**

**Excluded studies and reasons for exclusion.**

**Excluded studies:**

Studies were excluded primarily from the review for the following reasons: (a) out the scope of the prevalence of IPI (n=44); (b) not a representative sample of the general population (n=16); (c) narrative review (n=5).

**Excluded studies and reasons for exclusion.**

| Alasil SM, Abdullah KA. An epidemiological review on emerging and re-emerging parasitic infectious diseases in Malaysia. Open Microbiol J [Internet]. 2019;13(1):112–20. Available from: http://dx.doi.org/10.2174/1874285801913010112  **Reason for exclusion: review** |
| --- |
| Valenciano PA, Soriano I, Sisican HM, Paragas EF, Tabarina KT, Ramos KB, et al. The Prevalence of Soil-Transmitted Helminths (STH) and Entamoeba spp. Infections in Southeast Asia: A Systematic Review. Infections in Southeast Asia: A Systematic Review Asian Journal of Biological and Life Sciences. 2023;12(2).  **Reason for exclusion: review** |
| Lubna M. E. Amal R. N. Extremely low prevalence of intestinal cryptosporidiosis and hygienic practices among hospitalized children with malignancies in Malaysia: A preliminary observation. Afr J Microbiol Res [Internet]. 2011;5(27). Available from: http://dx.doi.org/10.5897/ajmr11.1117  **Reason for exclusion: out of scope** |
| Fregonesi BM, Suzuki MN, Machado CS, Tonani KA de A, Fernandes APM, Monroe AA, et al. Emergent and re-emergent parasites in HIV-infected children: immunological and socio-environmental conditions that are involved in the transmission of Giardia spp. and Cryptosporidium spp. Rev Soc Bras Med Trop [Internet]. 2015;48(6):753–8. Available from: http://dx.doi.org/10.1590/0037-8682-0119-2015  **Reason for exclusion: out of scope** |
| Sahimin N, Abu Bakar N, Lim YAL, Behnke JM, Lewis J, Kamaruddin N, et al. Entry of migrant workers to Malaysia: Consideration to implement mass drug administration against intestinal parasitic infections. Int J Health Policy Manag [Internet]. 2024; 13:7842. Available from: http://dx.doi.org/10.34172/ijhpm.2024.7842  **Reason for exclusion: out of scope** |
| Lim YAL, Nissapatorn V. Transmission of waterborne parasites in the Association of Southeast Asian Nations (ASEAN): Overview and direction forward. Food Waterborne Parasitol [Internet]. 2017;8–9:75–83. Available from: http://dx.doi.org/10.1016/j.fawpar.2017.08.001  **Reason for exclusion: out of scope** |
| Chan YL, Patterson CL, Priest JW, Stresman G, William T, Chua TH, et al. Assessing seroprevalence and associated risk factors for multiple infectious diseases in Sabah, Malaysia using serological multiplex bead assays. Front Public Health [Internet]. 2022;10:924316. Available from: http://dx.doi.org/10.3389/fpubh.2022.924316  **Reason for exclusion: out of scope** |
| Choy SH, Mahdy MAK, Al-Mekhlafi HM, Low VL, Surin J. Population expansion and gene flow in *Giardia duodenalis* as revealed by triosephosphate isomerase gene. Parasit Vectors [Internet]. 2015;8(1):454. Available from: http://dx.doi.org/10.1186/s13071-015-1084-y  **Reason for exclusion: out of scope** |
| Kumar T, Onichandran S, Lim YAL, Sawangjaroen N, Ithoi I, Andiappan H, et al. Comparative study on waterborne parasites between Malaysia and Thailand: A new insight. Am J Trop Med Hyg [Internet]. 2014;90(4):682–9. Available from: http://dx.doi.org/10.4269/ajtmh.13-0266  **Reason for exclusion: out of scope and not a representative sample of the general population** |
| Huey CS, Mahdy MAK, Al-Mekhlafi HM, Nasr NA, Lim YAL, Mahmud R, et al. Multilocus genotyping of *Giardia duodenalis* in Malaysia. Infect Genet Evol [Internet]. 2013;17:269–76. Available from: http://dx.doi.org/10.1016/j.meegid.2013.04.013  **Reason for exclusion: out of scope** |
| Lee SC, Ngui R, Tan TK, Roslan MA, Ithoi I, Mahdy MAK, et al. Understanding Giardia infections among rural communities using the one health approach. Acta Trop [Internet]. 2017; 176:349–54. Available from: http://dx.doi.org/10.1016/j.actatropica.2017.08.030  **Reason for exclusion: out of scope** |
| Nisha M, Talib NNA, Khir N, Daud IS, Davamani F. Knowledge, awareness, and practice (KAP) level of parasite infection among adults in Selangor, Malaysia. Int J Res Pharm Sci [Internet]. 2021;12(2):1632–8. Available from: http://dx.doi.org/10.26452/ijrps.v12i2.4753  **Reason for exclusion: out of scope** |
| Sinniah B, Hassan A KR, Sabaridah I, Soe MM, Ibrahim Z, Ali O. Prevalence of intestinal parasitic infections among communities living in different habitats and its comparison with one hundred and one studies conducted over the past 42 years (1970 to 2013) in Malaysia. Trop Biomed. 2014;31(2):190–206.  **Reason for exclusion: review** |
| Ngui R, Hassan N-A, Nordin NMS, Mohd-Shaharuddin N, Chang LY, Teh CSJ, et al. Copro-molecular study of Entamoeba infection among the indigenous community in Malaysia: A first report on the species-specific prevalence of Entamoeba in dogs. Acta Trop [Internet]. 2020;204(105334):105334. Available from: http://dx.doi.org/10.1016/j.actatropica.2020.105334  **Reason for exclusion: out of scope** |
| Ho JY, Lavinya AA, Kay DSW, Lee CIS, Razmi AH, Walsh CL, et al. Towards an integrated approach to improve the understanding of the relationships between water-borne infections and health outcomes: Using Malaysia as a detailed case study. Front Water [Internet]. 2022;4. Available from: http://dx.doi.org/10.3389/frwa.2022.779860  **Reason for exclusion: out of scope** |
| Mahmoudi M-R, Ongerth JE, Karanis P. Cryptosporidium and cryptosporidiosis: The Asian perspective. Int J Hyg Environ Health [Internet]. 2017;220(7):1098–109. Available from: http://dx.doi.org/10.1016/j.ijheh.2017.07.005  **Reason for exclusion: not a representative sample of the general population** |
| Izadi M, Jonaidi-Jafari N, Saburi A, Eyni H, Rezaiemanesh M-R, Ranjbar R. Prevalence, molecular characteristics and risk factors for cryptosporidiosis among Iranian immunocompromised patients: Cryptosporidiosis and immunodeficiency. Microbiol Immunol [Internet]. 2012;56(12):836–42. Available from: http://dx.doi.org/10.1111/j.1348-0421.2012.00513.x  **Reason for exclusion: not a representative sample of the general population** |
| Muhid A, Robertson I, Ng J, Ryan U. Prevalence of and management factors contributing to Cryptosporidium sp. infection in pre-weaned and post-weaned calves in Johor, Malaysia. Exp Parasitol [Internet]. 2011;127(2):534–8. Available from: http://dx.doi.org/10.1016/j.exppara.2010.10.015  **Reason for exclusion: out of scope** |
| Abdullah DA, Ola-Fadunsin SD, Ruviniyia K, Gimba FI, Chandrawathani P, Lim YAL, et al. Molecular detection and epidemiological risk factors associated with Cryptosporidium infection among cattle in Peninsular Malaysia. Food Waterborne Parasitol [Internet]. 2019;14(e00035):e00035. Available from: http://dx.doi.org/10.1016/j.fawpar.2019.e00035  **Reason for exclusion: out of scope** |
| Yusof AM, Isa ML. Knowledge, attitude and practices of intestinal helminths and protozoa infection among parents of school children in peripheral school and urban school area in Kuantan, Pahang, Malaysia. Journal of Biotechnology and Strategic Health Research. 2017;1(3):75–82.  **Reason for exclusion: out of scope** |
| Mohd-Qawiem F, Nur-Fazila SH, Ain-Fatin R, Yong QH, Nur-Mahiza MI, Yasmin AR. Detection of zoonotic-borne parasites in Rattus spp. in Klang Valley, Malaysia. Vet World [Internet]. 2022;15(4):1006–14. Available from: http://dx.doi.org/10.14202/vetworld.2022.1006-1014  **Reason for exclusion: out of scope** |
| Tahar AS, Bilung LM, Apun K, Richard RL, Hashim HF, Nillian E, et al. Contamination of waterborne parasites at water treatment plants and a gravity-feed system: A highlight on water safety for urban and rural communities in Kuching, Sarawak. Int J Biol Biomed Eng [Internet]. 2022;16:298–310. Available from: http://dx.doi.org/10.46300/91011.2022.16.37  **Reason for exclusion: out of scope** |
| Onichandran S, Kumar T, Lim YAL, Sawangjaroen N, Andiappan H, Salibay CC, et al. Waterborne parasites and physico-chemical assessment of selected lakes in Malaysia. Parasitol Res [Internet]. 2013;112(12):4185–91. Available from: http://dx.doi.org/10.1007/s00436-013-3610-1  **Reason for exclusion: out of scope** |
| Abdulwahab MH, Al-Talib H. Estimation of protozoal diversity among stray cats in Malaysia. Biodiversitas [Internet]. 2024;25(5). Available from: http://dx.doi.org/10.13057/biodiv/d250529  **Reason for exclusion: out of scope** |
| Ting Lo N, Abul Bashar Sarker M, Ai Lian Lim Y, Harun-Or-Rashid M, Sakamoto J. Inadequate water treatment quality as assessed by protozoa removal in Sarawak, Malaysia. Nagoya J Med Sci [Internet]. 2018;80(2):165–74. Available from: http://dx.doi.org/10.18999/nagjms.80.2.165  **Reason for exclusion: out of scope** |
| Sazalli HN, Kamaruzaman IN, Tarmizi MR, Okene IA, Shaari R, Bamaiyi PH. Ancylostomiasis, Giardiasis and Isosporiasis in a domestic short hair cat in Kota Bharu, Malaysia. J. Adv. Parasitol. 2016 Apr 22;3(3):75-80.  **Reason for exclusion: out of scope** |
| Ngui R, Lee SC, Yap NJ, Tan TK, Aidil RM, Chua KH, et al. Gastrointestinal parasites in rural dogs and cats in Selangor and Pahang states in Peninsular Malaysia. Acta Parasitol [Internet]. 2014;59(4):737–44. Available from: http://dx.doi.org/10.2478/s11686-014-0306-3  **Reason for exclusion: out of scope** |
| Tahar AS, Bilung LM, Apun K, Richard RL, Lim YAL. Epidemiological study of human intestinal parasites in Sarawak, East Malaysia: A review. Trop Biomed [Internet]. 2021;38(3):377–86. Available from: http://dx.doi.org/10.47665/tb.38.3.083  **Reason for exclusion: review** |
| Kumar T, Abd Majid MA, Onichandran S, Jaturas N, Andiappan H, Salibay CC, et al. Presence of *Cryptosporidium parvum* and *Giardia lamblia* in water samples from Southeast Asia: towards an integrated water detection system. Infect Dis Poverty [Internet]. 2016;5(1):3. Available from: http://dx.doi.org/10.1186/s40249-016-0095-z  **Reason for exclusion: out of scope** |
| Lee SC, Ngui R, Tan TK, Roslan MA, Ithoi I, Lim YAL. Aquatic biomonitoring of Giardia cysts and Cryptosporidium oocysts in peninsular Malaysia. Environ Sci Pollut Res Int [Internet]. 2014;21(1):445–53. Available from: http://dx.doi.org/10.1007/s11356-013-1925-1  **Reason for exclusion: out of scope** |
| Roshidi N, Mohd Hassan NH, Abdul Hadi A, Arifin N. Current state of infection and prevalence of giardiasis in Malaysia: a review of 20 years of research. PeerJ [Internet]. 2021;9(e12483):e12483. Available from: http://dx.doi.org/10.7717/peerj.12483  **Reason for exclusion: review** |
| Anuar TS, Al-Mekhlafi HM, Ghani MKA, Azreen SN, Salleh FM, Ghazali N, et al. First molecular identification of *Entamoeba moshkovskii* in Malaysia. Parasitology [Internet]. 2012;139(12):1521–5. Available from: http://dx.doi.org/10.1017/S0031182012001485  **Reason for exclusion: out of scope** |
| Zaheer T, Imran M, Abbas RZ, Zaheer I, Malik MA. Avian cryptosporidiosis and its zoonotic significance in Asia. Worlds Poult Sci J [Internet]. 2021;77(1):55–70. Available from: http://dx.doi.org/10.1080/00439339.2020.1866961  **Reason for exclusion: out of scope** |
| Mat Yusof A, Hashim N, Md Isa ML. First molecular identification of Cryptosporidium by 18S rRNA in goats and association with farm management in Terengganu. Asian Pac J Trop Biomed [Internet]. 2017;7(5):385–8. Available from: http://dx.doi.org/10.1016/j.apjtb.2017.01.008  **Reason for exclusion: out of scope** |
| Quah JX, Ambu S, Lim YAL, Mahdy MAK, Mak JW. Molecular identification of *Cryptosporidium parvum* from avian hosts. Parasitology [Internet]. 2011;138(5):573–7. Available from: http://dx.doi.org/10.1017/S0031182010001691  **Reason for exclusion: out of scope** |
| Tengku SA, Norhayati M. Public health and clinical importance of amoebiasis in Malaysia: a review. Trop Biomed. 2011;28(2):194–222.  **Reason for exclusion: out of scope** |
| Wong W-K, Mohd-Nor N, Noordin R, Foo P-C, Mohamed Z, Haq JA, et al. Parasitic infections in Malaysian aborigines with pulmonary tuberculosis: a comparative cross-sectional study. Parasitol Res [Internet]. 2019;118(9):2635–42. Available from: http://dx.doi.org/10.1007/s00436-019-06406-7  **Reason for exclusion: out of scope** |
| Mohammed Mahdy AK, Lim YAL, Surin J, Wan KL, Al-Mekhlafi MSH. Risk factors for endemic giardiasis: highlighting the possible association of contaminated water and food. Trans R Soc Trop Med Hyg [Internet]. 2008;102(5):465–70. Available from: http://dx.doi.org/10.1016/j.trstmh.2008.02.004  **Reason for exclusion: out of scope** |
| Othman N, Mohamed Z, Verweij JJ, Huat LB, Olivos-García A, Yeng C, et al. Application of real-time polymerase chain reaction in detection of *Entamoeba histolytica* in pus aspirates of liver abscess patients. Foodborne Pathog Dis [Internet]. 2010;7(6):637–41. Available from: http://dx.doi.org/10.1089/fpd.2009.0427  **Reason for exclusion: out of scope** |
| Tan ZN, Wong WK, Nik Zairi Z, Abdullah B, Rahmah N, Zeehaida M, et al. Identification of *Entamoeba histolytica* trophozoites in fresh stool sample: comparison of three staining techniques and study on the viability period of the trophozoites. Trop Biomed. 2010;27(1):79–88.  **Reason for exclusion: out of scope** |
| Alyousefi NA, Mahdy MAK, Mahmud R, Lim YAL. Factors associated with high prevalence of intestinal protozoan infections among patients in Sana’a City, Yemen. PLoS One [Internet]. 2011;6(7): e22044. Available from: http://dx.doi.org/10.1371/journal.pone.0022044  **Reason for exclusion: not a representative sample of the general population** |
| Sejdini A, Mahmud R, Lim YAL, Mahdy M, Sejdini F, Gjoni V, et al. Intestinal parasitic infections among children in central Albania. Ann Trop Med Parasitol [Internet]. 2011;105(3):241–50. Available from: http://dx.doi.org/10.1179/136485911X12987676649584  **Reason for exclusion: not a representative sample of the general population** |
| Iqbal A, Lim YAL, Surin J, Sim BLH. High diversity of Cryptosporidium subgenotypes identified in Malaysian HIV/AIDS individuals targeting gp60 gene. PLoS One [Internet]. 2012;7(2):e31139. Available from: http://dx.doi.org/10.1371/journal.pone.0031139  **Reason for exclusion: out of scope** |
| Rayani M, Unyah NZ, Hatam GR, Abdullah WO, Hamat RA. Prevalence and molecular genotypic characterization of *Giardia duodenalis* isolates from Iranian patients (Fars province). Int J Infect Dis [Internet]. 2012;16:e173. Available from: http://dx.doi.org/10.1016/j.ijid.2012.05.720  **Reason for exclusion: not a representative sample of the general population** |
| Siti Shafiyyah CO, Jamaiah I, Rohela M, Lau YL, Siti Aminah F. Prevalence of intestinal and blood parasites among wild rats in Kuala Lumpur, Malaysia. Trop Biomed. 2012;29(4):544–50.  **Reason for exclusion: out of scope** |
| Anuar TS, Al-Mekhlafi HM, Abdul Ghani MK, Abu Bakar E, Azreen SN, Salleh FM, et al. Evaluation of formalin-ether sedimentation and trichrome staining techniques: its effectiveness in detecting *Entamoeba histolytica/dispar/moshkovskii* in stool samples. J Microbiol Methods [Internet]. 2013;92(3):344–8. Available from: http://dx.doi.org/10.1016/j.mimet.2013.01.010  **Reason for exclusion: out of scope** |
| Mohammad KN, Badrul MM, Mohamad N, Zainal-Abidin AH. Protozoan parasites of four species of wild anurans from a local zoo in Malaysia. Trop Biomed. 2013;30(4):615–20.  **Reason for exclusion: out of scope** |
| Aniesona AT, Bamaiyi PH. Retrospective study of cryptosporidiosis among diarrhoeic children in the arid region of north-eastern Nigeria. Zoonoses Public Health [Internet]. 2014;61(6):420–6. Available from: http://dx.doi.org/10.1111/zph.12088  **Reason for exclusion: not a representative sample of the general population** |
| Mohd Shah NA, Wan Abdul Wahab WNA, Mohd Nawi SFA, Mohd-Zain Z, Latif B, Suhaimi R. Comparison of the Anaerocult A and the oil blocking methods for the in vitro cultivation of *Entamoeba histolytica*. Malays J Pathol. 2015;37(3):271–4.  **Reason for exclusion: out of scope** |
| Sungkar S, Pohan APN, Ramadani A, Albar N, Azizah F, Nugraha ARA, et al. Heavy burden of intestinal parasite infections in Kalena Rongo village, a rural area in South West Sumba, eastern part of Indonesia: a cross sectional study. BMC Public Health [Internet]. 2015;15(1):1296. Available from: http://dx.doi.org/10.1186/s12889-015-2619-z  **Reason for exclusion: not a representative sample of the general population** |
| Chuah CJ, Mukhaidin N, Choy SH, Smith GJD, Mendenhall IH, Lim YAL, et al. Prevalence of Cryptosporidium and Giardia in the water resources of the Kuang River catchment, Northern Thailand. Sci Total Environ [Internet]. 2016;562:701–13. Available from: http://dx.doi.org/10.1016/j.scitotenv.2016.03.247  **Reason for exclusion: not a representative sample of the general population** |
| Richard RL, Ithoi I, Abd Majid MA, Wan Sulaiman WY, Tan TC, Nissapatorn V, et al. Monitoring of waterborne parasites in two drinking water treatment plants: A study in Sarawak, Malaysia. Int J Environ Res Public Health [Internet]. 2016;13(7):641. Available from: http://dx.doi.org/10.3390/ijerph13070641  **Reason for exclusion: out of scope** |
| Hisamuddin NH, Hashim N, Soffian SN, Amin MHM, Wahab RA, Mohammad M, et al. Identification of Cryptosporidium from dairy cattle in Pahang, Malaysia. Korean J Parasitol [Internet]. 2016;54(2):197–200. Available from: http://dx.doi.org/10.3347/kjp.2016.54.2.197  **Reason for exclusion: out of scope** |
| Maria DGS, Irene MR, Fransiska M, Rizqiani AK, Yayuk Hartriyanti E, Elsa Herdiana Murhandarwati E. Prevalence of intestinal protozoan infections and association with hygiene knowledge among primary schoolchildren in Salahutu and Leihitu districts, Central Maluku regency, Indonesia. Trop Biomed. 2016;33(3):428–36.  **Reason for exclusion: not a representative sample of the general population** |
| Yap NJ, Koehler AV, Ebner J, Tan TK, Lim YAL, Gasser RB. Molecular analysis of Cryptosporidium from cattle from five states of Peninsular Malaysia. Mol Cell Probes [Internet]. 2016;30(1):39–43. Available from: http://dx.doi.org/10.1016/j.mcp.2016.01.002  **Reason for exclusion: out of scope** |
| Al-Areeqi MA, Sady H, Al-Mekhlafi HM, Anuar TS, Al-Adhroey AH, Atroosh WM, et al. First molecular epidemiology of *Entamoeba histolytica, E. dispar* and *E. moshkovskii* infections in Yemen: different species-specific associated risk factors. Trop Med Int Health [Internet]. 2017;22(4):493–504. Available from: http://dx.doi.org/10.1111/tmi.12848  **Reason for exclusion: not a representative sample of the general population** |
| Al-Mekhlafi HM. *Giardia duodenalis* infection among rural communities in Yemen: A community-based assessment of the prevalence and associated risk factors. Asian Pac J Trop Med [Internet]. 2017;10(10):987–95. Available from: http://dx.doi.org/10.1016/j.apjtm.2017.09.011  **Reason for exclusion: not a representative sample of the general population** |
| Bilung LM, Tahar AS, Yunos NE, Apun K, Lim YA-L, Nillian E, et al. Detection of Cryptosporidium and Cyclospora oocysts from environmental water for drinking and recreational activities in Sarawak, Malaysia. Biomed Res Int [Internet]. 2017;2017:4636420. Available from: http://dx.doi.org/10.1155/2017/4636420  **Reason for exclusion: out of scope** |
| Shrivastava AK, Kumar S, Mohakud NK, Suar M, Sahu PS. Multiple etiologies of infectious diarrhea and concurrent infections in a pediatric outpatient-based screening study in Odisha, India. Gut Pathog [Internet]. 2017;9(1):16. Available from: http://dx.doi.org/10.1186/s13099-017-0166-0  **Reason for exclusion: not a representative sample of the general population** |
| Zheng H, He J, Wang L, Zhang R, Ding Z, Hu W. Risk factors and spatial clusters of Cryptosporidium infection among school-age children in a rural region of Eastern China. Int J Environ Res Public Health [Internet]. 2018;15(5):924. Available from: http://dx.doi.org/10.3390/ijerph15050924  **Reason for exclusion: not a representative sample of the general population** |
| Muslim A, Mohd Sofian S, Shaari SA, Hoh B-P, Lim YA-L. Prevalence, intensity and associated risk factors of soil transmitted helminth infections: A comparison between Negritos (indigenous) in inland jungle and those in resettlement at town peripheries. PLoS Negl Trop Dis [Internet]. 2019;13(4):e0007331. Available from: http://dx.doi.org/10.1371/journal.pntd.0007331  **Reason for exclusion: out of scope** |
| Tan TK, Low VL, Ng WH, Ibrahim J, Wang D, Tan CH, et al. Occurrence of zoonotic Cryptosporidium and *Giardia duodenalis* species/genotypes in urban rodents. Parasitol Int [Internet]. 2019;69:110–3. Available from: http://dx.doi.org/10.1016/j.parint.2018.12.007  **Reason for exclusion: out of scope** |
| Latifah U, Supargiyono, Subronto YW, Septiani L. The relationship between intestinal parasitic infection and CD4+ level among HIV patients in DR. Sardjito Central Hospital, Yogyakarta. In: Proceedings Of The 3rd International Seminar on Metallurgy and Materials (ISMM2019): Exploring New Innovation in Metallurgy and Materials. AIP Publishing; 2020.  **Reason for exclusion: out of scope** |
| Nhidza AF, Naicker T, Stray-Pedersen B, Chisango TJ, Sibanda EP, Ismail A, et al. Immune response to asymptomatic infections by *Entamoeba histolytica* and other enteric pathogens in pregnant women and their infants in a high HIV burdened setting in Zimbabwe. J Microbiol Immunol Infect [Internet]. 2020;53(4):612–21. Available from: http://dx.doi.org/10.1016/j.jmii.2018.11.005  **Reason for exclusion: not a representative sample of the general population** |
| Saidu AS, Mohammed S, Adamu SG, Sadiq MA, Tijjani AO, Musa HI, et al. Prevalence and risk estimates of Cryptosporidium oocysts infection associated with consumption of raw-eaten vegetables in Maiduguri metropolis LGAs, Northeast Nigeria. Sci Rep [Internet]. 2023;13(1):23079. Available from: http://dx.doi.org/10.1038/s41598-023-49451-0  **Reason for exclusion: not a representative sample of the general population** |
