# Supplementary material for: Prevalence and risk factors of intestinal protozoal infections among patients in Malaysia: A systematic review and meta-analysis
Source: PLoS One. 2025 Sep 11;20(9):e0332218. doi: 10.1371/journal.pone.0332218 (PMC12425333; doi:10.1371/journal.pone.0332218)
Supplement: S4 Appendix — (DOCX) [file pone.0332218.s004.docx]

**S4 APPENDIX**

**Sub-Group Forest Plot.**


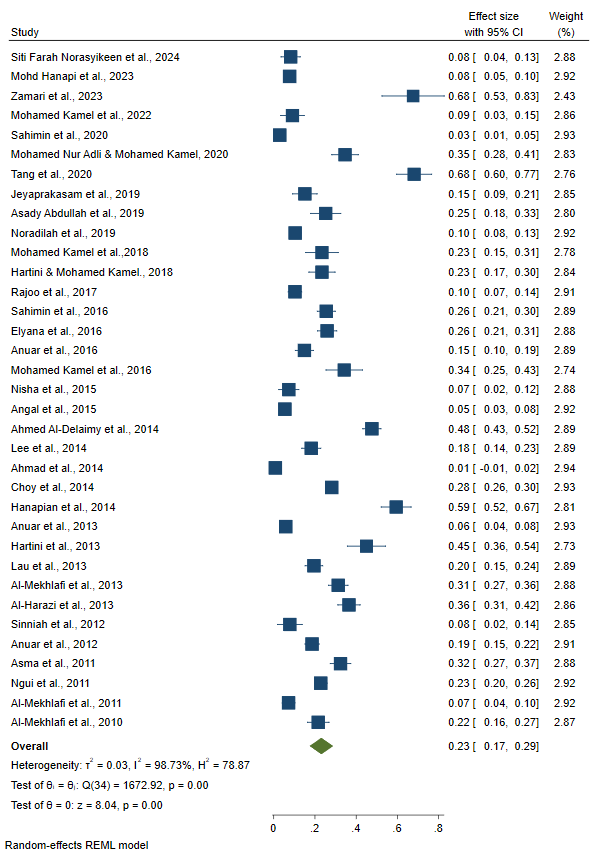


Fig S4.1 Forest plot of current relative frequency of intestinal protozoa among patients in Malaysia studies diagnosed by microscopy method.


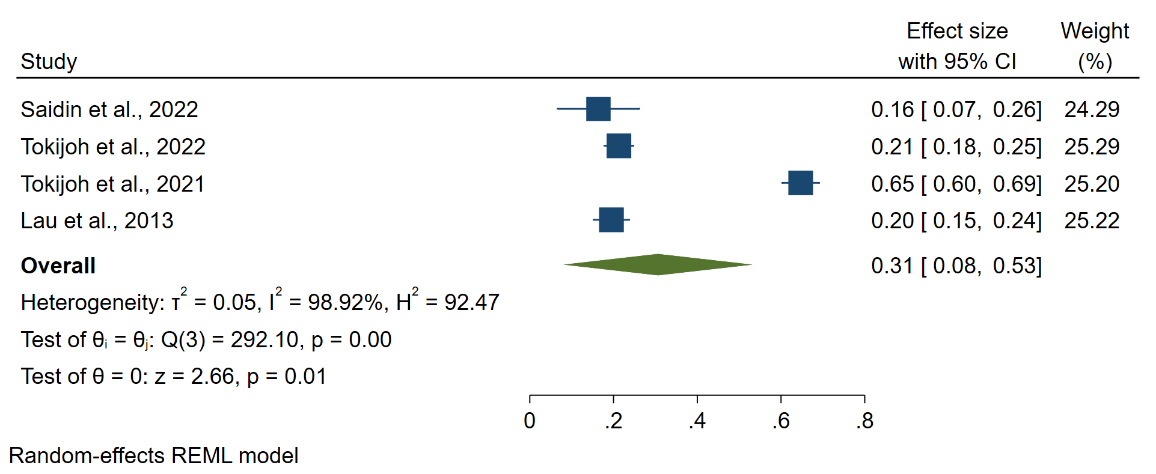


Fig S4.2 Forest plot of current relative frequency of intestinal protozoa among patients in Malaysia studies diagnosed by molecular method.


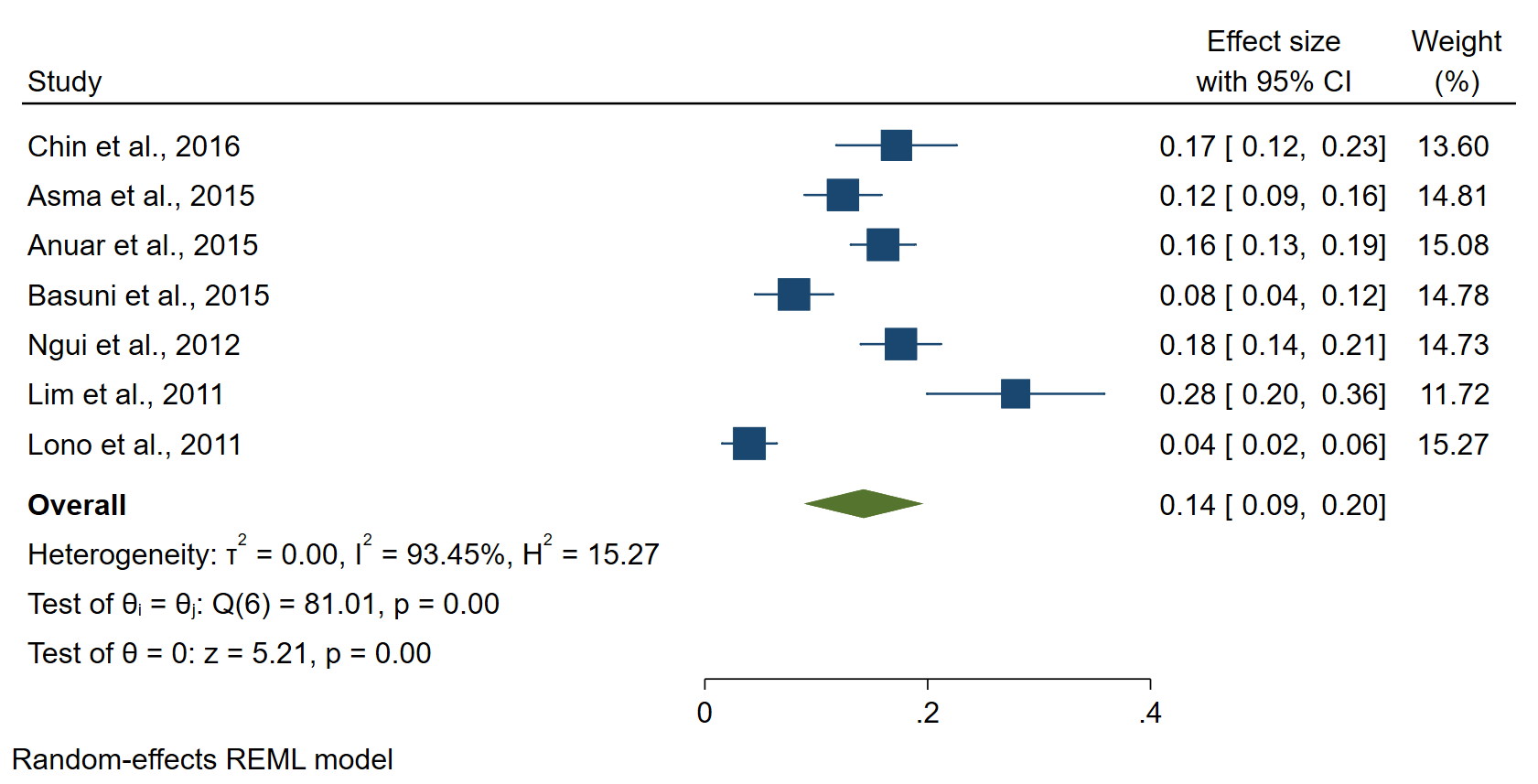


Fig S4.3 Forest plot of current relative frequency of intestinal protozoa among patients in Malaysia studies diagnosed by microscopy and molecular method.


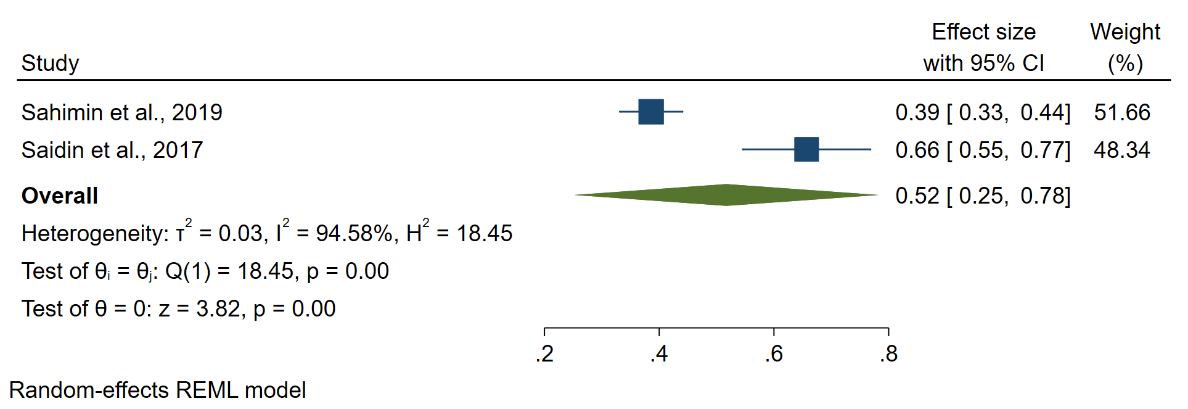


Fig S4.4 Forest plot of current relative frequency of intestinal protozoa among patients in Malaysia studies diagnosed by microscopy, molecular and immunoassay combined.


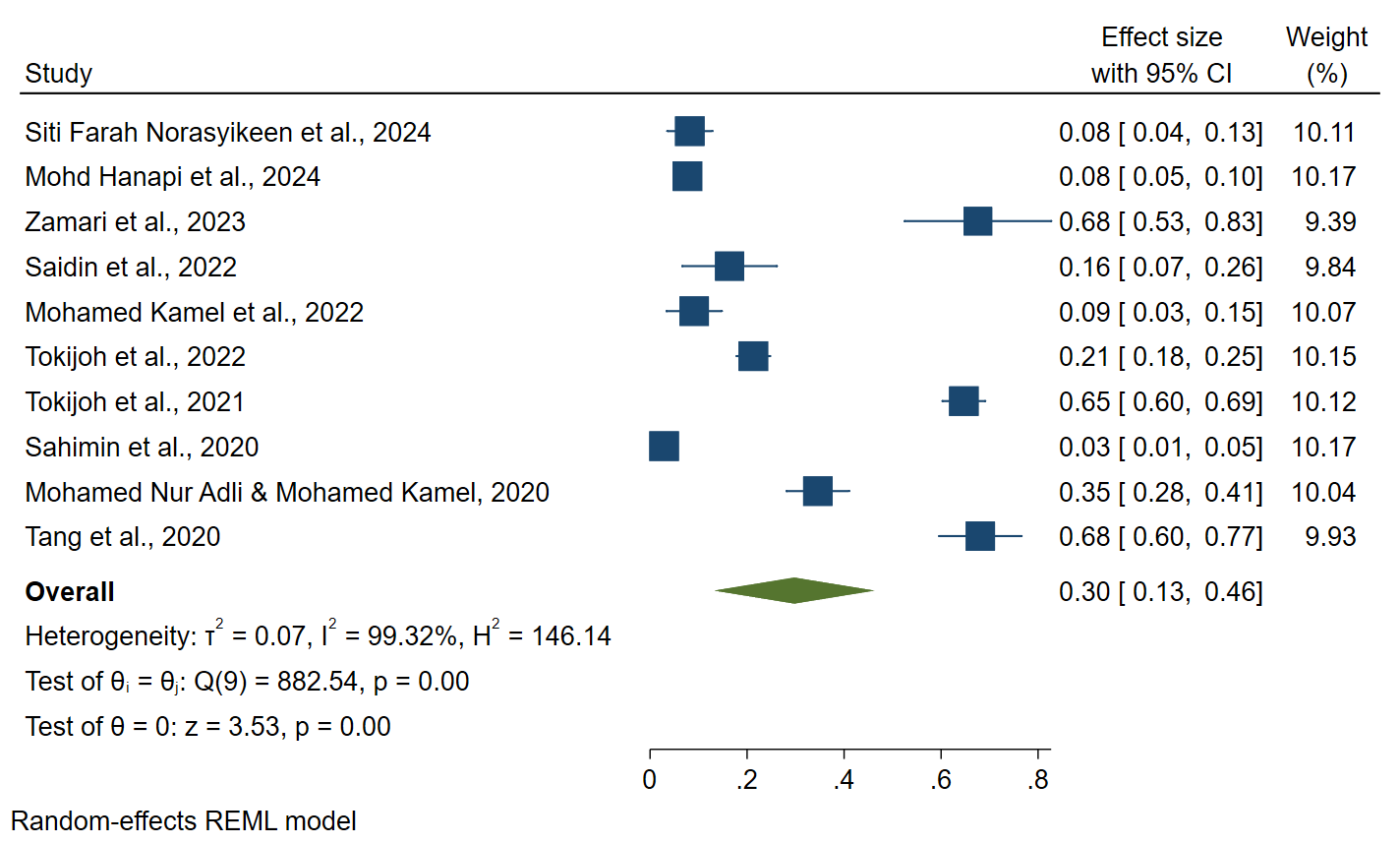


Fig S4.5 Forest plot of current relative frequency of intestinal protozoa among patients in Malaysia studies that report based on the year of 2020-2024.


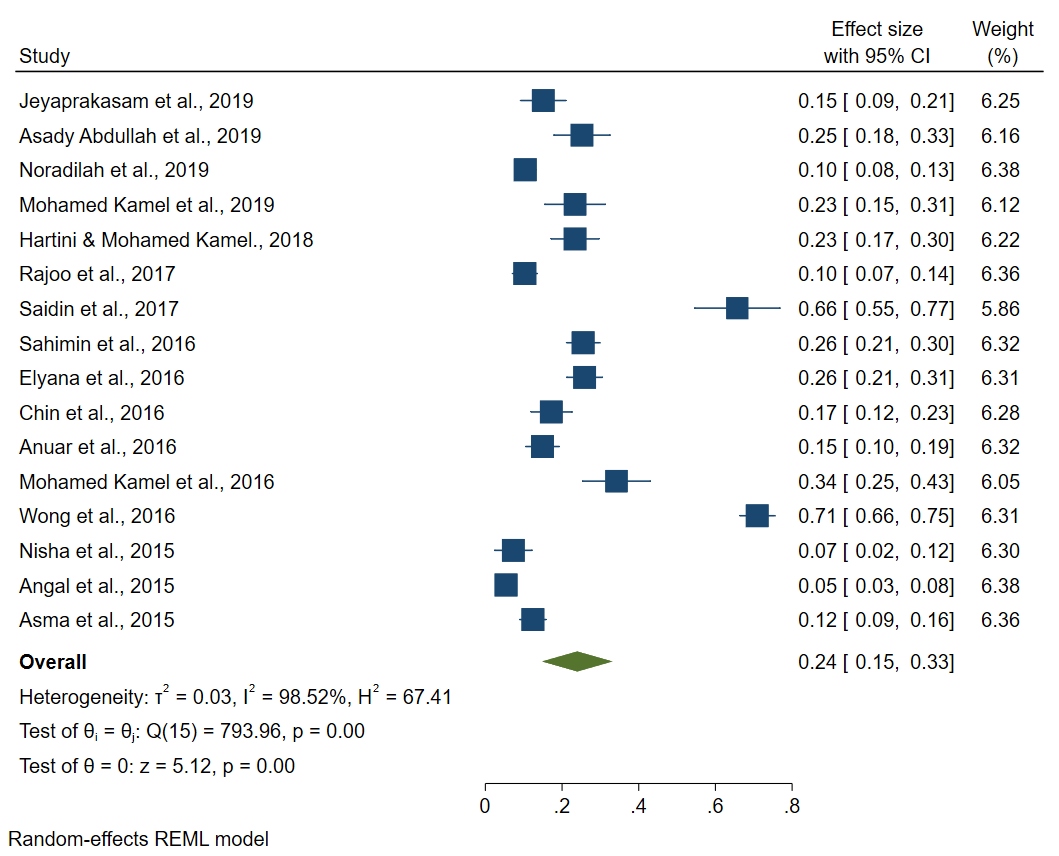


Fig S4.6 Forest plot of current relative frequency of intestinal protozoa among patients in Malaysia studies that report based on the year of 2015-2019.


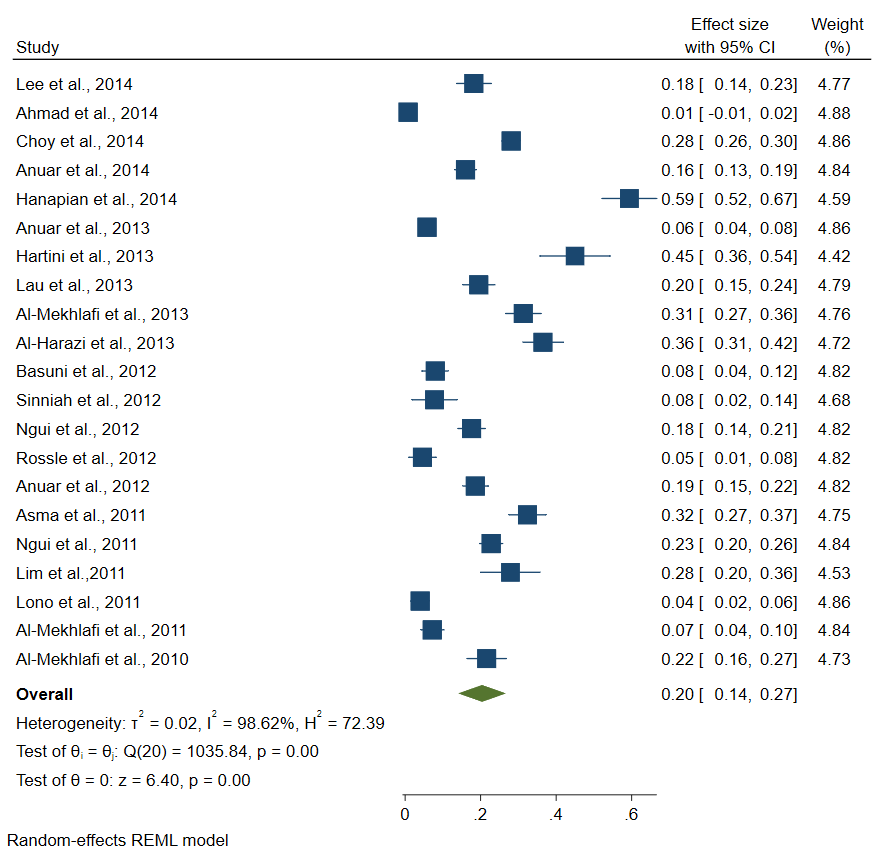


Fig S4.7 Forest plot of current relative frequency of intestinal protozoa among patients in Malaysia studies that report based on the year of 2010-2015.


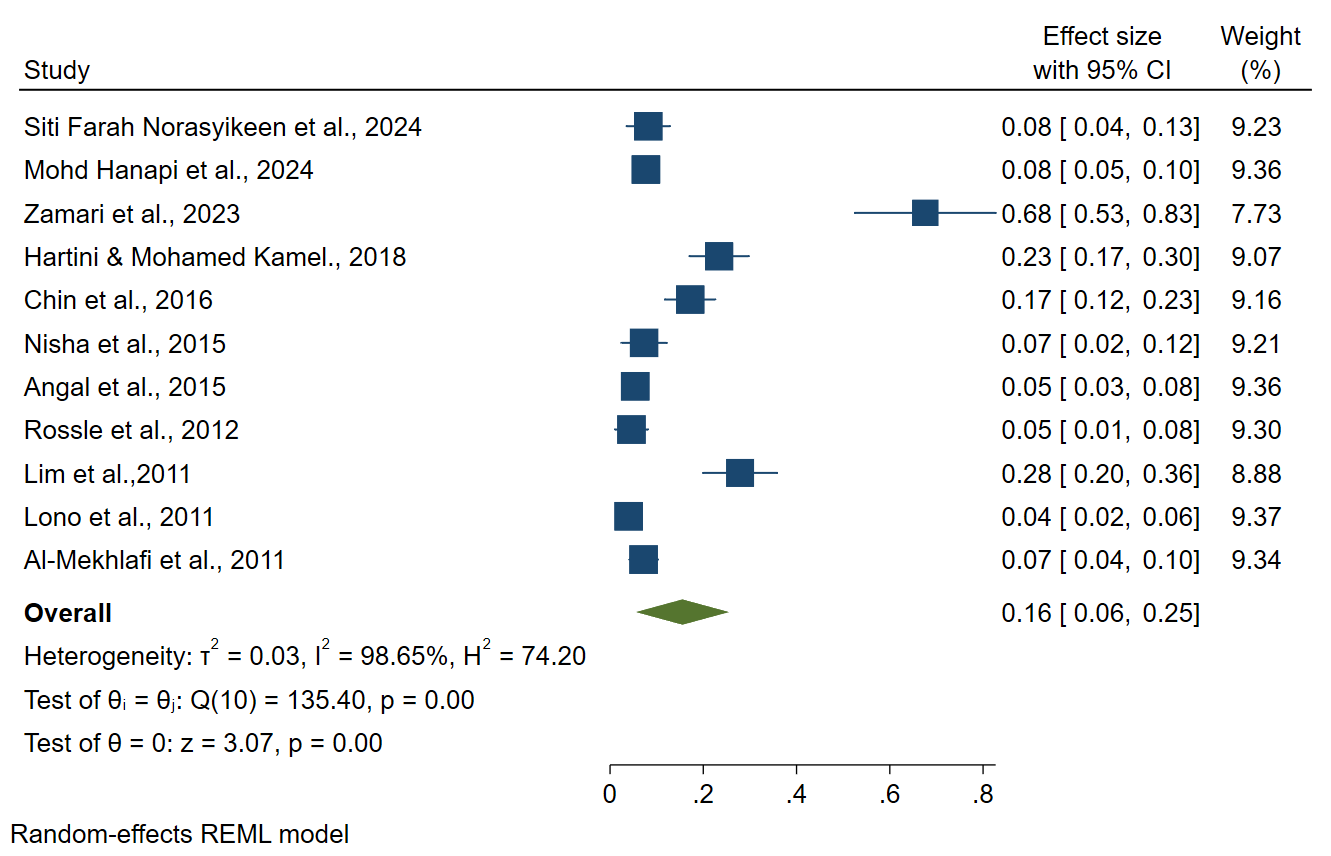


Fig S4.8 Forest plot of current relative frequency of intestinal protozoa among patients in Selangor and KL.


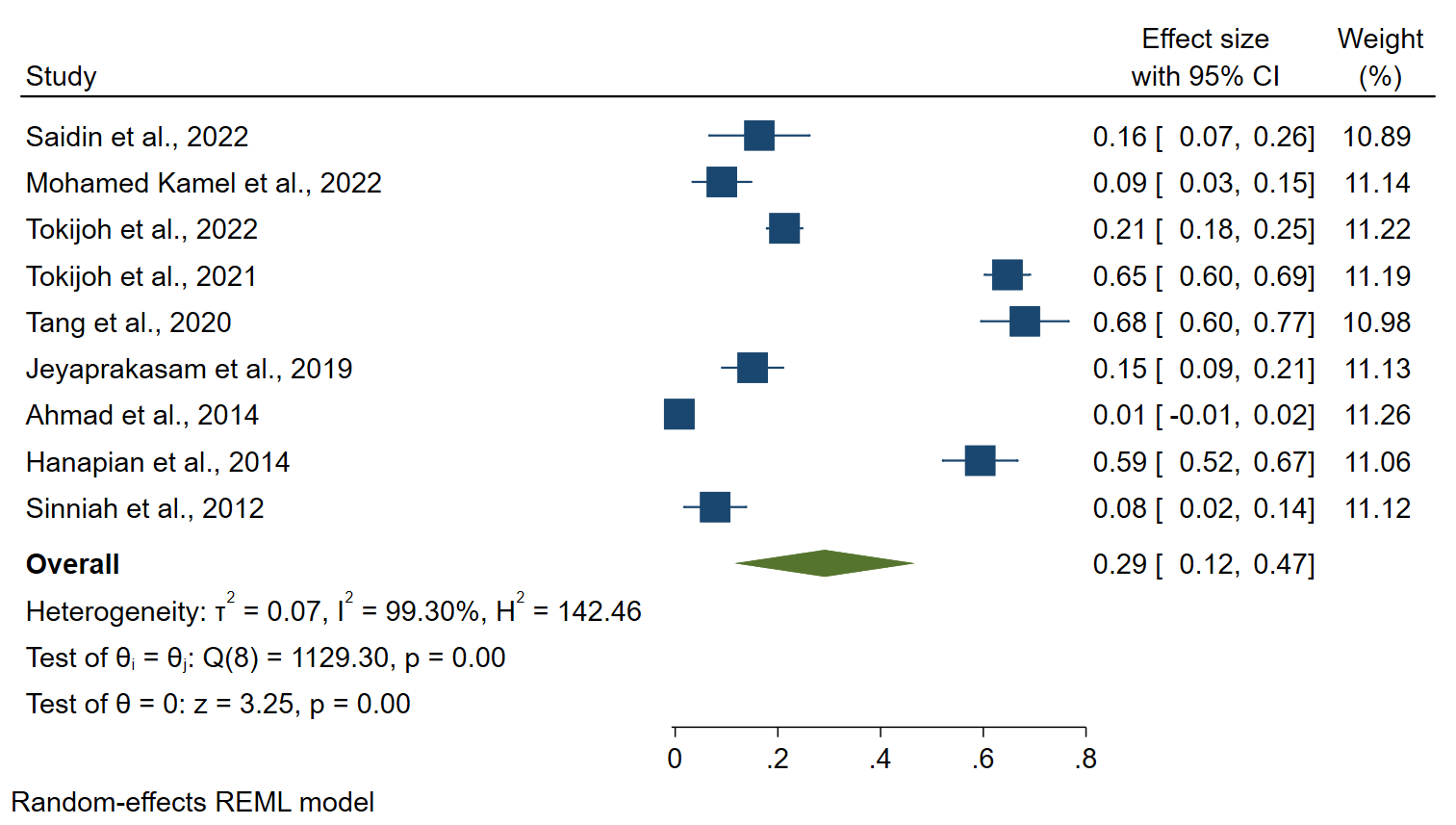


Fig S4.9 Forest plot of current relative frequency of intestinal protozoa among patients in Perak.


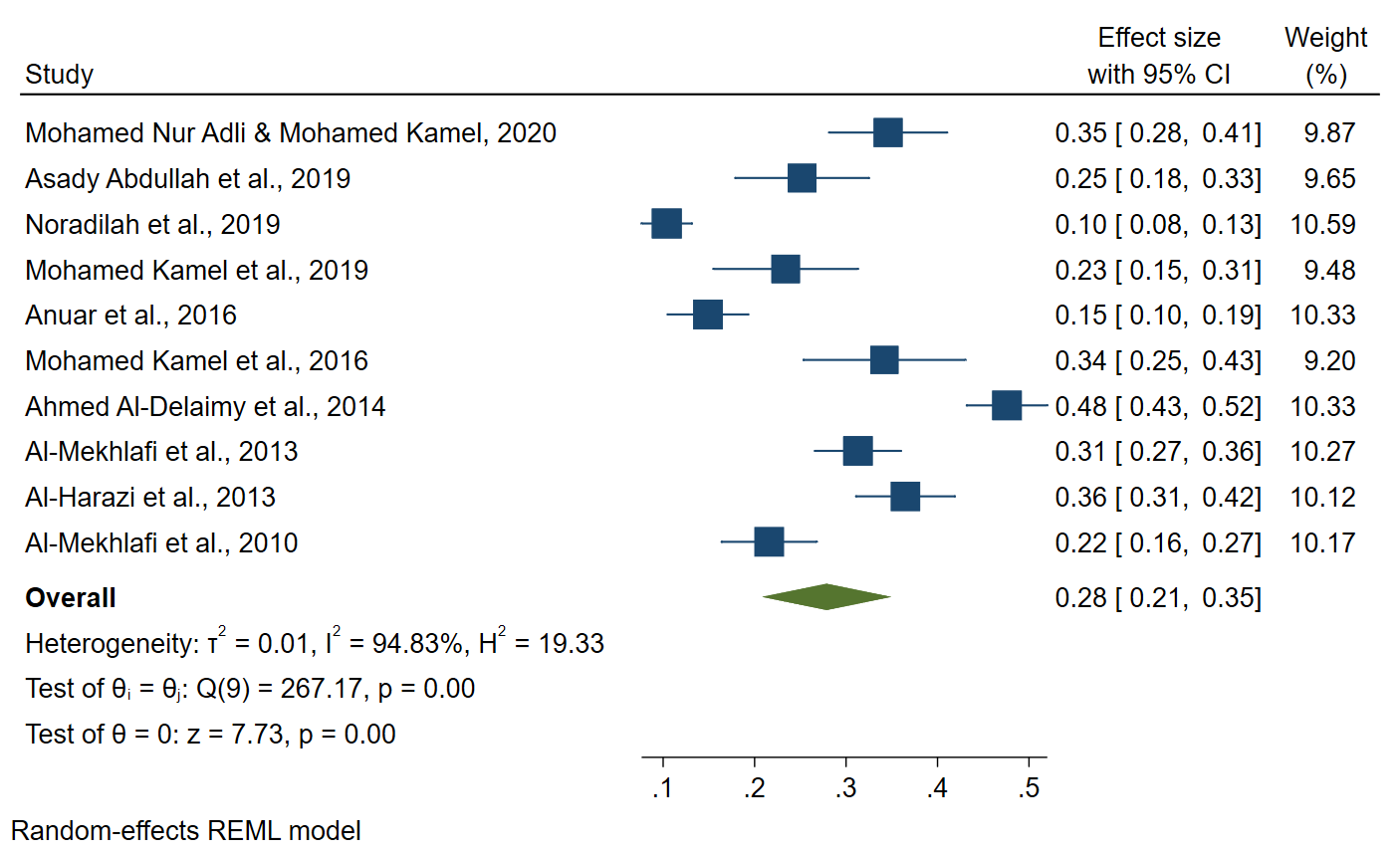


Fig S4.10 Forest plot of current relative frequency of intestinal protozoa among patients in Pahang.


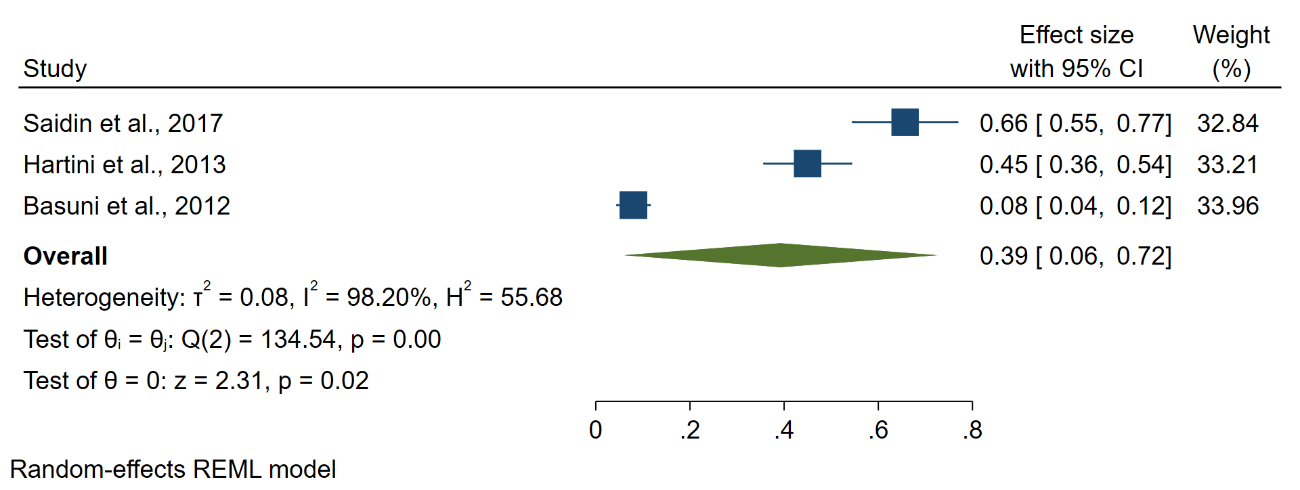


Fig S4.11 Forest plot of current relative frequency of intestinal protozoa among patients in Kelantan.


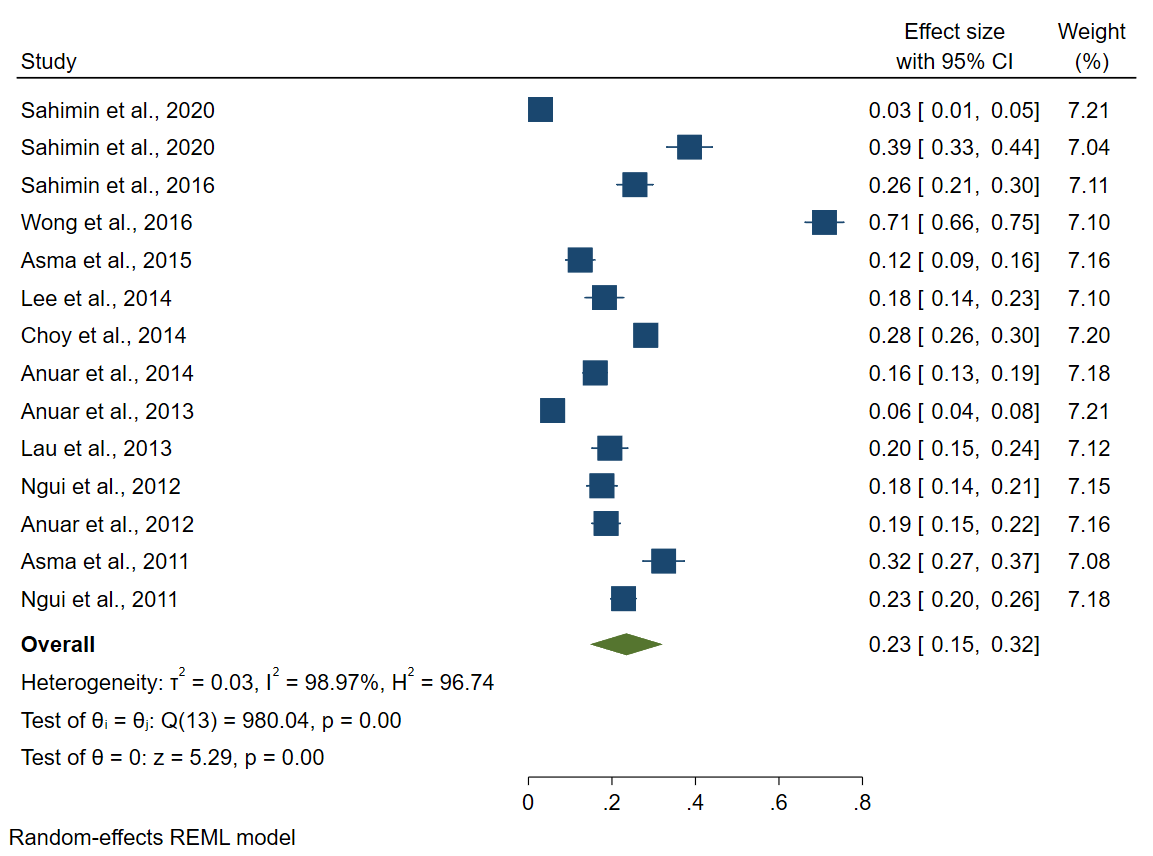


Fig S4.12 Forest plot of current relative frequency of intestinal protozoa among patients in Peninsular Malaysia.


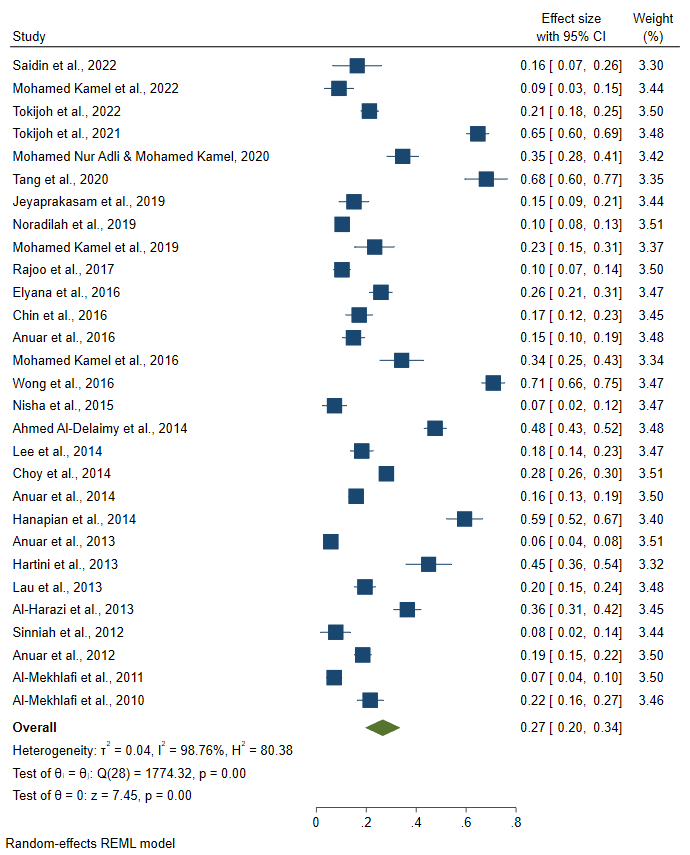


Fig S4.13 Forest plot of current relative frequency of intestinal protozoa among aboriginal communities in Malaysia.


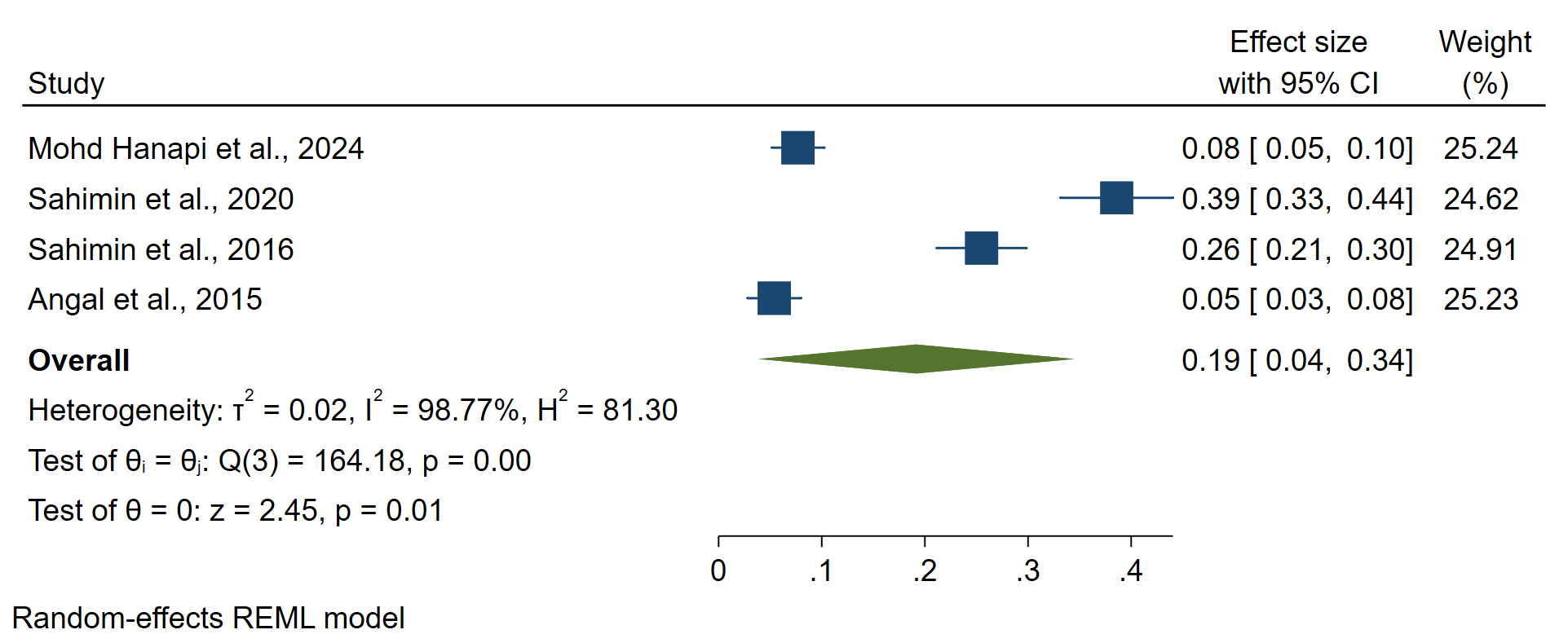


Fig S4.14 Forest plot of current relative frequency of intestinal protozoa among migrants in Malaysia.


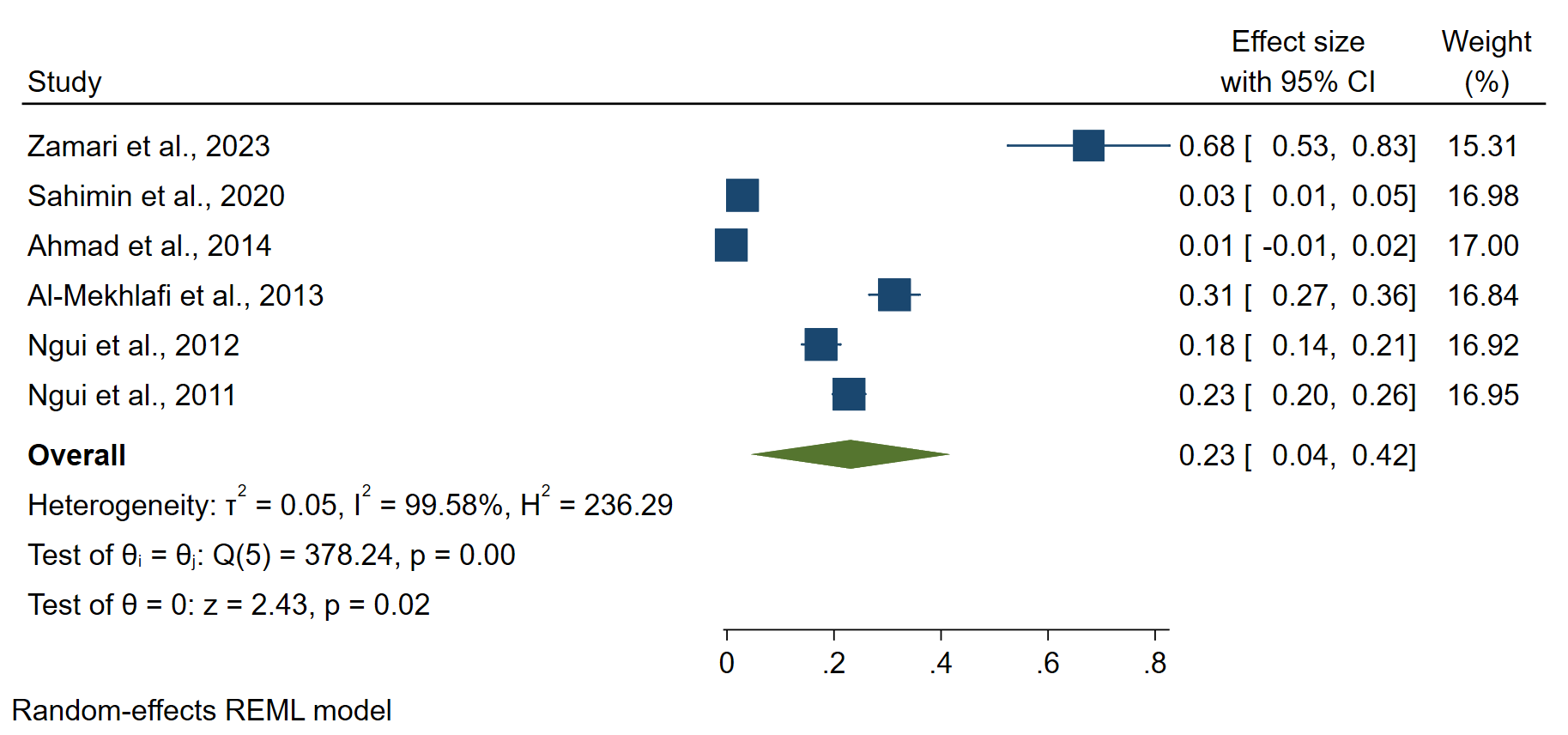
Fig S4.15 Forest plot of current relative frequency of intestinal protozoa local communities in Malaysia.


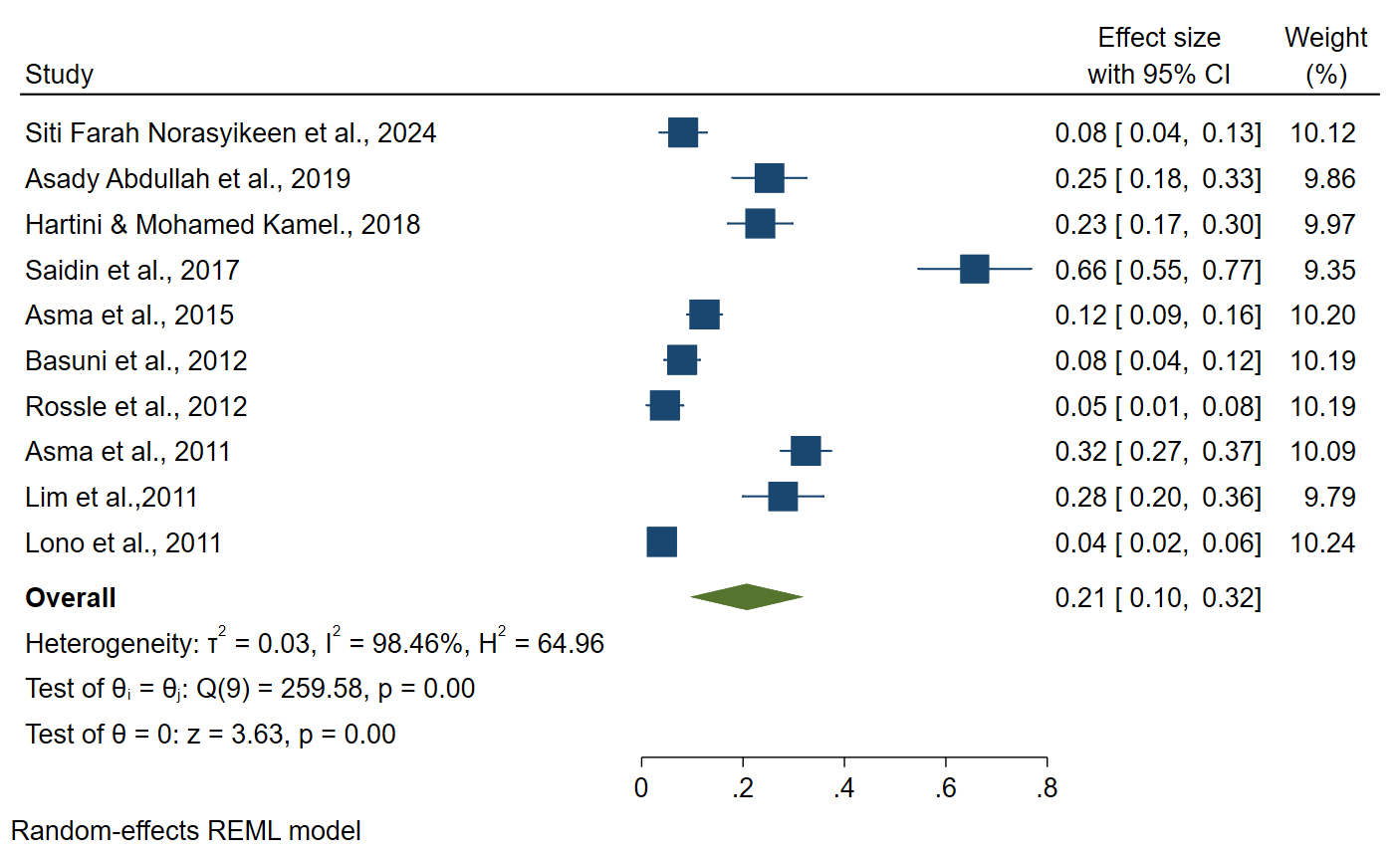


Fig S4.16 Forest plot of current relative frequency of intestinal protozoa hospitalised patients in Malaysia.
