# Supplementary material for: Prevalence and risk factors of intestinal protozoal infections among patients in Malaysia: A systematic review and meta-analysis
Source: PLoS One. 2025 Sep 11;20(9):e0332218. doi: 10.1371/journal.pone.0332218 (PMC12425333; doi:10.1371/journal.pone.0332218)
Supplement: S5 Appendix — (DOCX) [file pone.0332218.s005.docx]

**S5 APPENDIX**

**Risk of bias assessment.**

The research articles selected for this review were assessed by an independent reviewer (NSM) and checked for methodological validity by the senior supervising author (HAT) before being included in the review. The Mun et al. tool for risk of bias in prevalence studies [1] was used (Appendix S). The advantage of this tool is that it distinguishes between the assessment of whether the research was conducted to the highest possible standards of methodological quality and the extent to which the results are at risk of bias. The Mun et al. instrument consists of nine questions, where a “yes" response implies a low risk of bias and a “no" response implies a high risk of bias. When interpreting studies on the prevalence of IPI, these important questions were considered in the present review. Published studies were screened and assessed, and studies that met the inclusion criteria were included in the systematic review.

*Joanna Briggs Institute’s critical appraisal checklist [1] for studies of prevalence data:*

| **No** | **Criteria** | **Response** |
| --- | --- | --- |
|  | Was the sample frame appropriate to address the target population? | *Yes, No, Unclear, Not applicable* |
| 2. | Were the study participants sampled in an appropriate way? | *Yes, No, Unclear, Not applicable* |
| 3. | Was the sample size adequate? | *Yes, No, Unclear, Not applicable* |
| 4. | Were the study subjects and the setting described in detail? | *Yes, No, Unclear, Not applicable* |
| 5. | Was the data analysis conducted with sufficient coverage of the identified sample? | *Yes, No, Unclear, Not applicable* |
| 6. | Were valid methods used for the identification of the condition? | *Yes, No, Unclear, Not applicable* |
| 7. | Was the condition measured in a standard, reliable way for all participants? | *Yes, No, Unclear, Not applicable* |
| 8. | Was there appropriate statistical analysis? | *Yes, No, Unclear, Not applicable* |
| 9. | Was the response rate adequate, and if not, was the low response rate managed appropriately? | *Yes, No, Unclear, Not applicable* |

**REFERENCES**

1. Munn Z, Moola S, Lisy K, Riitano D, Tufanaru C. Methodological guidance for systematic reviews of observational epidemiological studies reporting prevalence and cumulative incidence data. *Int J Evid Based Healthc*. 2015;*13*(3):147-53.
