# Supplementary material for: Prevalence and risk factors of intestinal protozoal infections among patients in Malaysia: A systematic review and meta-analysis
Source: PLoS One. 2025 Sep 11;20(9):e0332218. doi: 10.1371/journal.pone.0332218 (PMC12425333; doi:10.1371/journal.pone.0332218)
Supplement: S6 Appendix — (DOCX) [file pone.0332218.s006.docx]

**S6 APPENDIX**

**Risk bias assessment results.**

Table 1. A detailed of the risk bias assessment results for included published studies.

| Author (Year) | Q1 | Q2 | Q3 | Q4 | Q5 | Q6 | Q7 | Q8 | Q9 | Total Score | Quality Rating |
| --- | --- | --- | --- | --- | --- | --- | --- | --- | --- | --- | --- |
| Siti Farah Norasyikeen et al., 2024 | Yes | Yes | Yes | Yes | Yes | Yes | Yes | Yes | Yes | 0 | Low risk |
| Mohd Hanapi et al., 2024 | Yes | Yes | Yes | Yes | Yes | Yes | Yes | Yes | Yes | 0 | Low risk |
| Zamari et al., 2023 | No | Yes | No | Yes | No | Yes | Yes | Yes | Yes | 3 | Low risk |
| Saidin et al., 2022 | Yes | Yes | Yes | Yes | Yes | Yes | Yes | Yes | Yes | 0 | Low risk |
| Mohamed Kamel et al., 2022 | No | No | No | Yes | No | Yes | Yes | No | Yes | 4 | Moderate risk |
| Tokijoh et al., 2022 | Yes | Yes | Yes | Yes | Yes | Yes | Yes | Yes | Yes | 0 | Low risk |
| Tokijoh et al., 2021 | Yes | Yes | Yes | Yes | Yes | Yes | Yes | Yes | Yes | 0 | Low risk |
| Sahimin et al., 2020 | Yes | Yes | Yes | Yes | Yes | Yes | Yes | Yes | Yes | 0 | Low risk |
| Adli & Mohamed Kamel, 2020 | Yes | Yes | Yes | Yes | No | Yes | No | No | No | 4 | Moderate risk |
| Tang et al., 2020 | Yes | Yes | Yes | No | No | Yes | No | No | Yes | 4 | Moderate risk |
| Sahimin et al., 2020 | Yes | Yes | Yes | Yes | Yes | Yes | Yes | Yes | Yes | 5 | Low risk |
| Jeyaprakasam et al., 2019 | Yes | Yes | Yes | Yes | No | No | Yes | No | No | 5 | Moderate risk |
| Asady Abdullah et al., 2019 | Yes | Yes | Yes | Yes | Yes | Yes | Yes | Yes | Yes | 0 | Low risk |
| Noradilah et al., 2019 | Yes | Yes | Yes | Yes | Yes | Yes | Yes | Yes | Yes | 0 | Low risk |
| Mohamed Kamel et al., 2019 | Yes | Yes | Yes | No | No | Yes | No | No | Yes | 4 | Medium risk |
| Hartini & Mohamed Kamel., 2018 | Yes | Yes | Yes | No | No | Yes | Yes | No | No | 4 | Moderate risk |
| Rajoo et al., 2017 | Yes | Yes | Yes | Yes | Yes | Yes | Yes | Yes | Yes | 0 | Low risk |
| Saidin et al., 2017 | Yes | Yes | Yes | Yes | Yes | Yes | Yes | Yes | Yes | 0 | Low risk |
| Sahimin et al., 2016 | Yes | Yes | Yes | Yes | Yes | Yes | Yes | Yes | Yes | 0 | Low risk |
| Elyana et al., 2016 | Yes | Yes | Yes | Yes | Yes | Yes | Yes | Yes | Yes | 0 | Low risk |
| Chin et al., 2016 | Yes | Yes | Yes | Yes | Yes | Yes | Yes | Yes | Yes | 0 | Low risk |
| Anuar et al., 2016 | Yes | Yes | Yes | No | No | Yes | Yes | No | No | 4 | Moderate risk |
| Mohamed Kamel et al., 2016 | Yes | Yes | Yes | No | No | Yes | Yes | No | No | 4 | Moderate risk |
| Wong et al., 2016 | Yes | Yes | Yes | Yes | Yes | Yes | Yes | Yes | Yes | 0 | Low risk |
| Nisha et al., 2015 | Yes | Yes | Yes | No | No | Yes | Yes | No | Yes | 4 | Moderate risk |
| Angal et al., 2015 | Yes | Yes | Yes | Yes | Yes | Yes | Yes | Yes | Yes | 0 | Low risk |
| Asma et al., 2015 | Yes | Yes | Yes | Yes | Yes | Yes | Yes | Yes | Yes | 0 | Low risk |
| Ahmed Al-Delaimy et al., 2014 | Yes | Yes | Yes | Yes | Yes | Yes | Yes | Yes | Yes | 0 | Low risk |
| Lee et al., 2014 | Yes | Yes | Yes | Yes | Yes | Yes | Yes | Yes | Yes | 0 | Low risk |
| Ahmad et al., 2014 | Yes | Yes | Yes | Yes | No | No | No | No | Yes | 4 | Moderate risk |
| Choy et al., 2014 | Yes | Yes | Yes | Yes | Yes | Yes | Yes | Yes | Yes | 0 | Low risk |
| Anuar et al., 2014 | Yes | Yes | Yes | Yes | Yes | Yes | Yes | Yes | No | 0 | Low risk |
| Hanapian et al., 2014 | Yes | Yes | Yes | No | No | Yes | Yes | No | No | 4 | Moderate risk |
| Anuar et al., 2013 | Yes | Yes | Yes | Yes | Yes | Yes | Yes | Yes | Yes | 0 | Low risk |
| Hartini et al., 2013 | Yes | Yes | Yes | No | No | Yes | Yes | No | No | 4 | Moderate risk |
| Lau et al., 2013 | Yes | Yes | Yes | Yes | Yes | Yes | Yes | Yes | Yes | 0 | Low risk |
| Al-Mekhlafi et al., 2013 | Yes | Yes | Yes | Yes | Yes | Yes | Yes | Yes | Yes | 0 | Low risk |
| Al-Harazi et al., 2013 | Yes | Yes | Yes | No | No | Yes | Yes | No | No | 4 | Moderate risk |
| Basuni et al., 2012 | Yes | Yes | Yes | Yes | Yes | Yes | Yes | Yes | Yes | 0 | Low risk |
| Sinniah et al., 2012 | Yes | Yes | No | Yes | No | Yes | Yes | No | No | 5 | Moderate risk |
| Ngui et al., 2012 | Yes | Yes | Yes | Yes | Yes | Yes | Yes | Yes | Yes | 0 | Low risk |
| Rossle et al., 2012 | Yes | Yes | Yes | No | Yes | Yes | Yes | Yes | Yes | 4 | Moderate risk |
| Anuar et al., 2012 | Yes | Yes | Yes | Yes | Yes | Yes | Yes | Yes | Yes | 0 | Low risk |
| Asma et al., 2011 | Yes | Yes | No | No | No | Yes | Yes | Yes | Yes | 2 | Low risk |
| Ngui et al., 2011 | Yes | Yes | Yes | Yes | Yes | Yes | Yes | Yes | Yes | 0 | Low risk |
| Lim et al.,2011 | Yes | Yes | Yes | Yes | Yes | Yes | Yes | Yes | Yes | 0 | Low risk |
| Lono et al., 2011 | Yes | Yes | Yes | Yes | Yes | Yes | Yes | Yes | Yes | 0 | Low risk |
| Al-Mekhlafi et al., 2011 | Yes | Yes | Yes | No | No | No | Yes | No | No | 4 | Medium risk |
| Al-Mekhlafi et al., 2010 | Yes | Yes | Yes | No | No | Yes | Yes | No | No | 4 | Medium risk |

Q= Question, Y= Yes (0 point), N=No (1 point), Low risk=0 to 3 points, Moderate risk= 4 to 6 points, High risk= 7 to 9 points.

The risk bias assessment of the included published studies was low in 32 studies (66.6%), moderate in 17 studies (34.7%), and there was no high-risk of bias across studies.
