# Supplementary material for: Prevalence and risk factors of intestinal protozoal infections among patients in Malaysia: A systematic review and meta-analysis
Source: PLoS One. 2025 Sep 11;20(9):e0332218. doi: 10.1371/journal.pone.0332218 (PMC12425333; doi:10.1371/journal.pone.0332218)
Supplement: S7 Appendix — (DOCX). [file pone.0332218.s007.docx]

**S7 APPENDIX**

**Sensitivity analysis forest plot after removing outlier.**


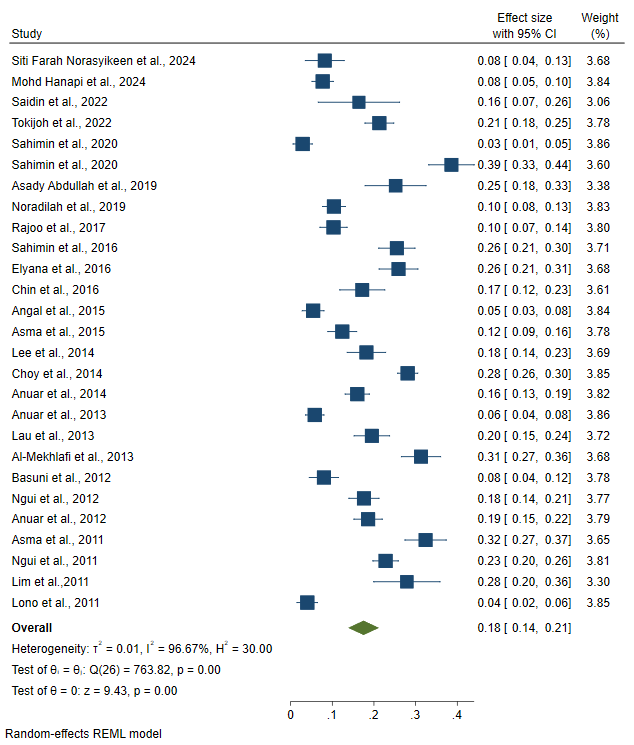


Fig S7.1 Sensitivity analysis for overall pooled prevalence for IPI after removing outlier.


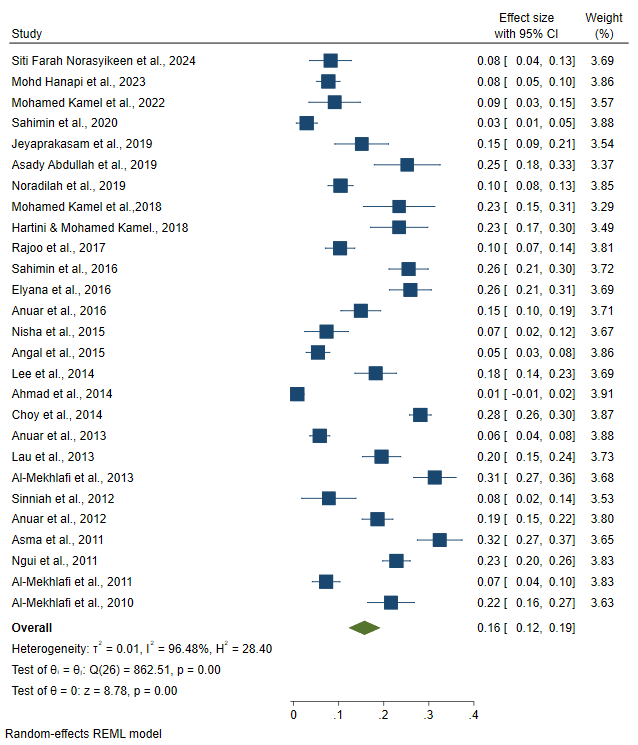


Fig S7. 2 Sensitivity analysis for microscopy method after removing outlier.


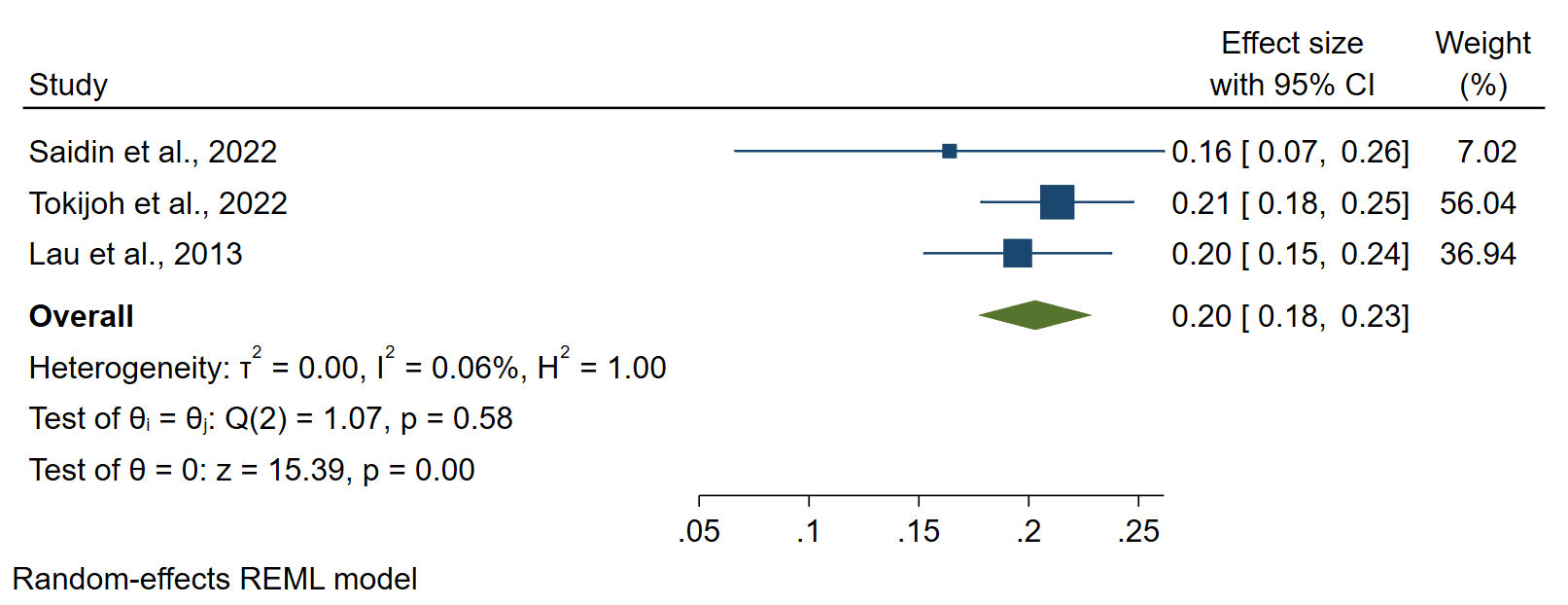


Fig S7. 3 Sensitivity analysis for molecular method after removing outlier.


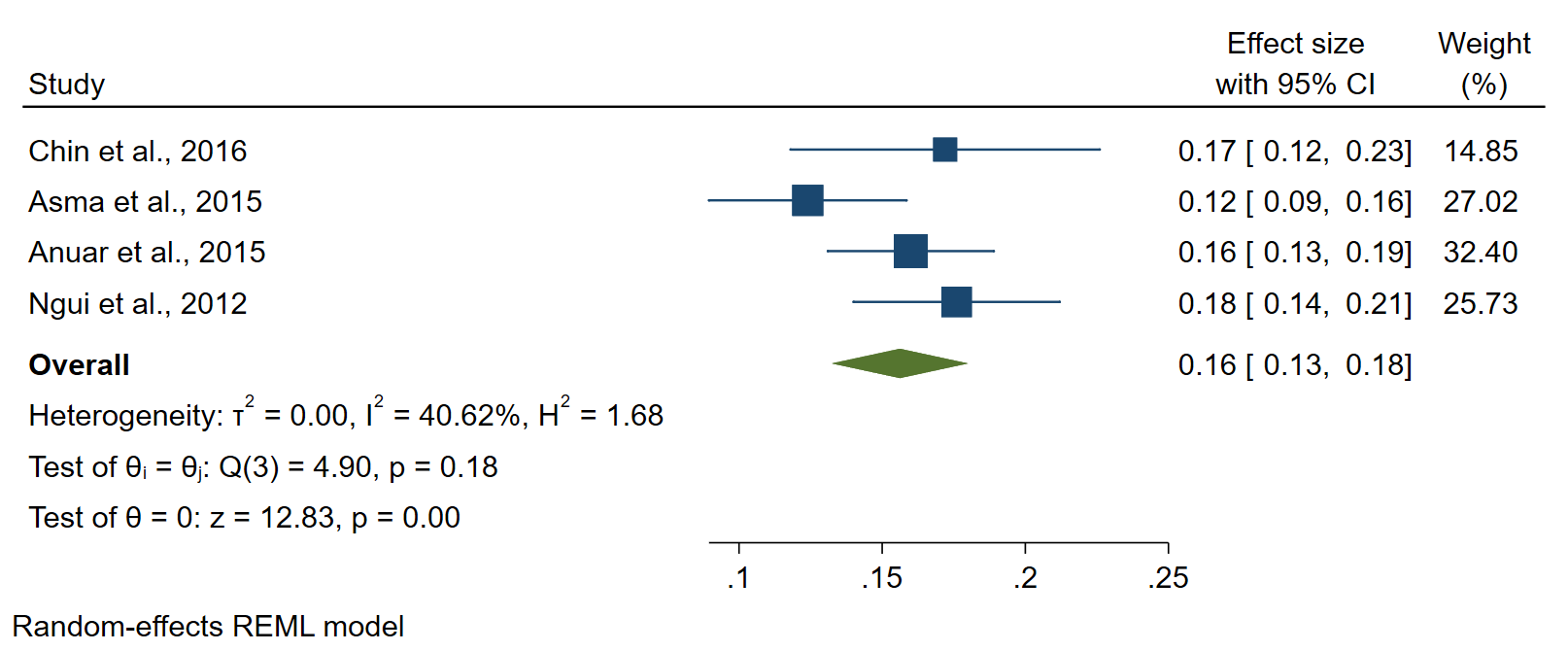


Fig S7.4 Sensitivity analysis for combination of microscopy and molecular method after removing outlier.


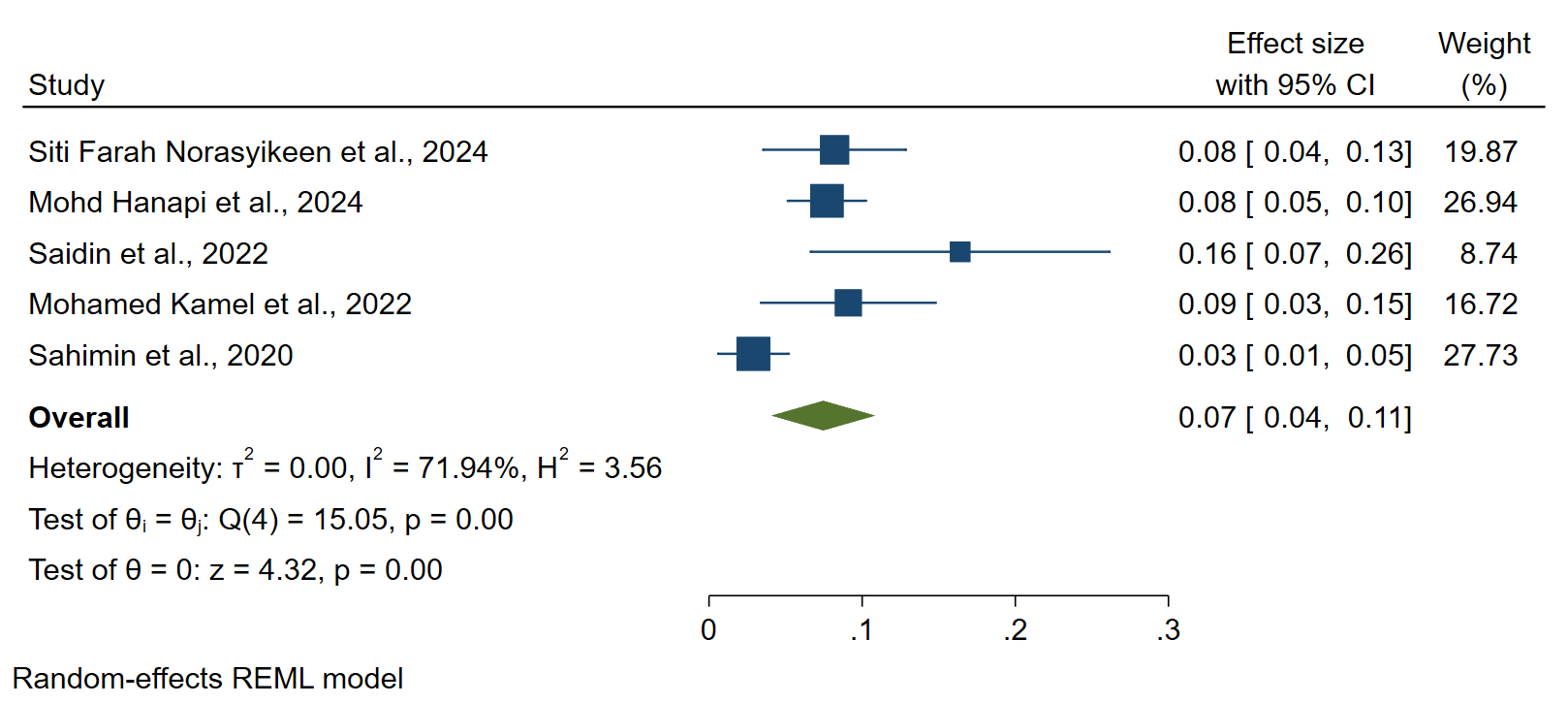


Fig S7.3 Sensitivity analysis for study year from 2020-2024 after removing outlier.


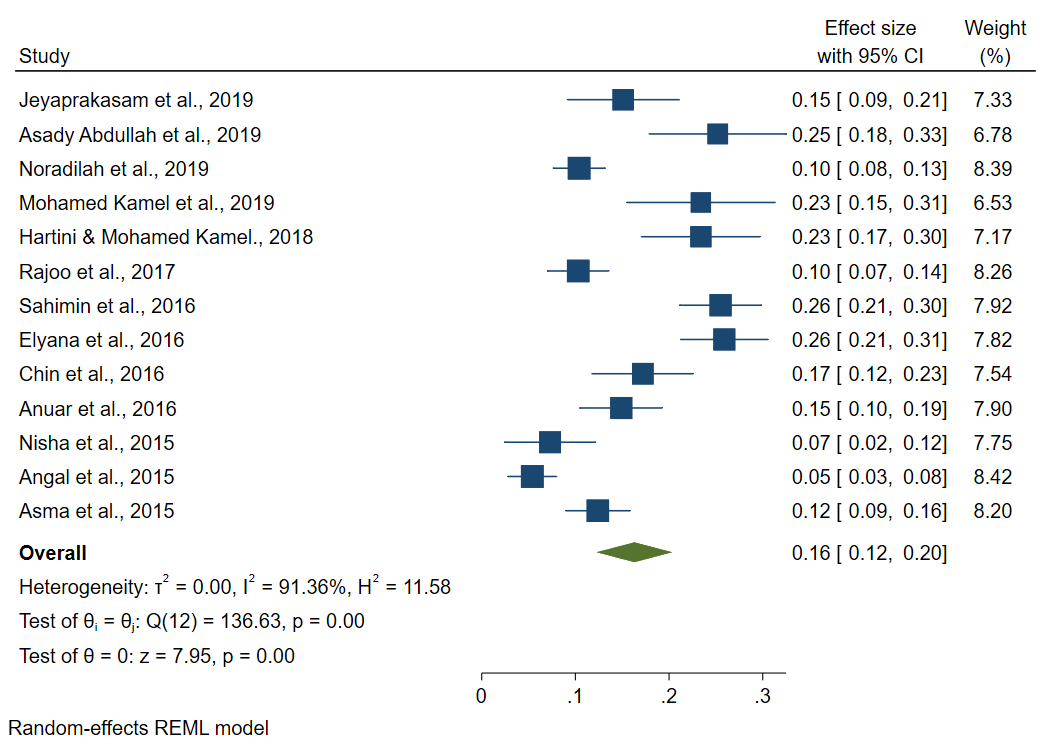


Fig S7.6 Sensitivity analysis for study year from 2015-2019 after removing outlier.


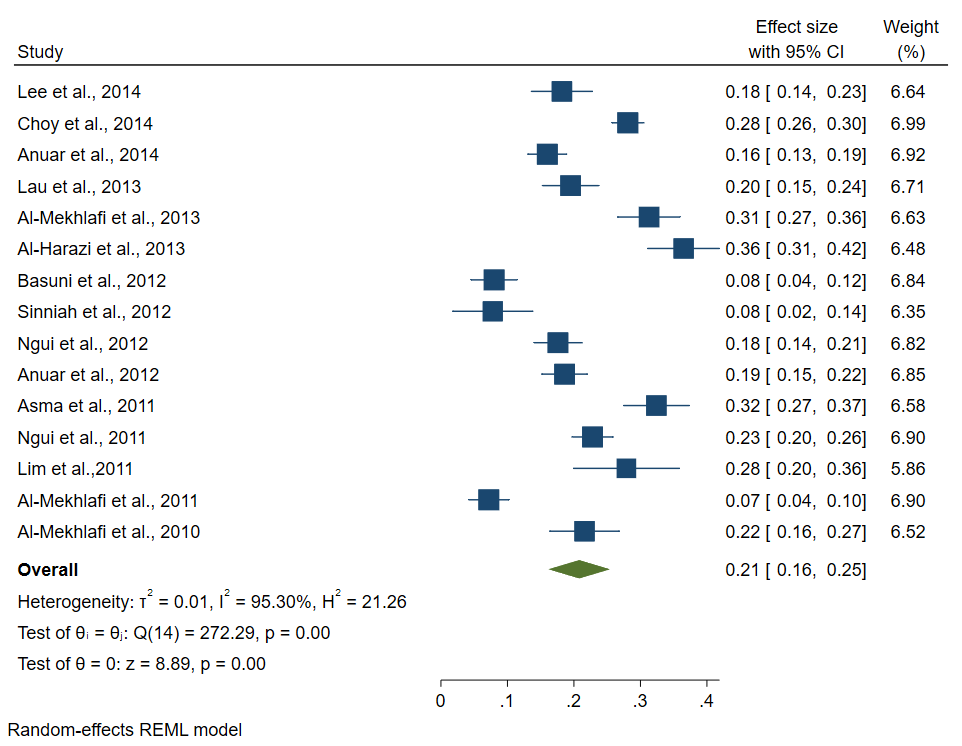


Fig S7.7 Sensitivity analysis for study year from 2010-2014 after removing outlier.


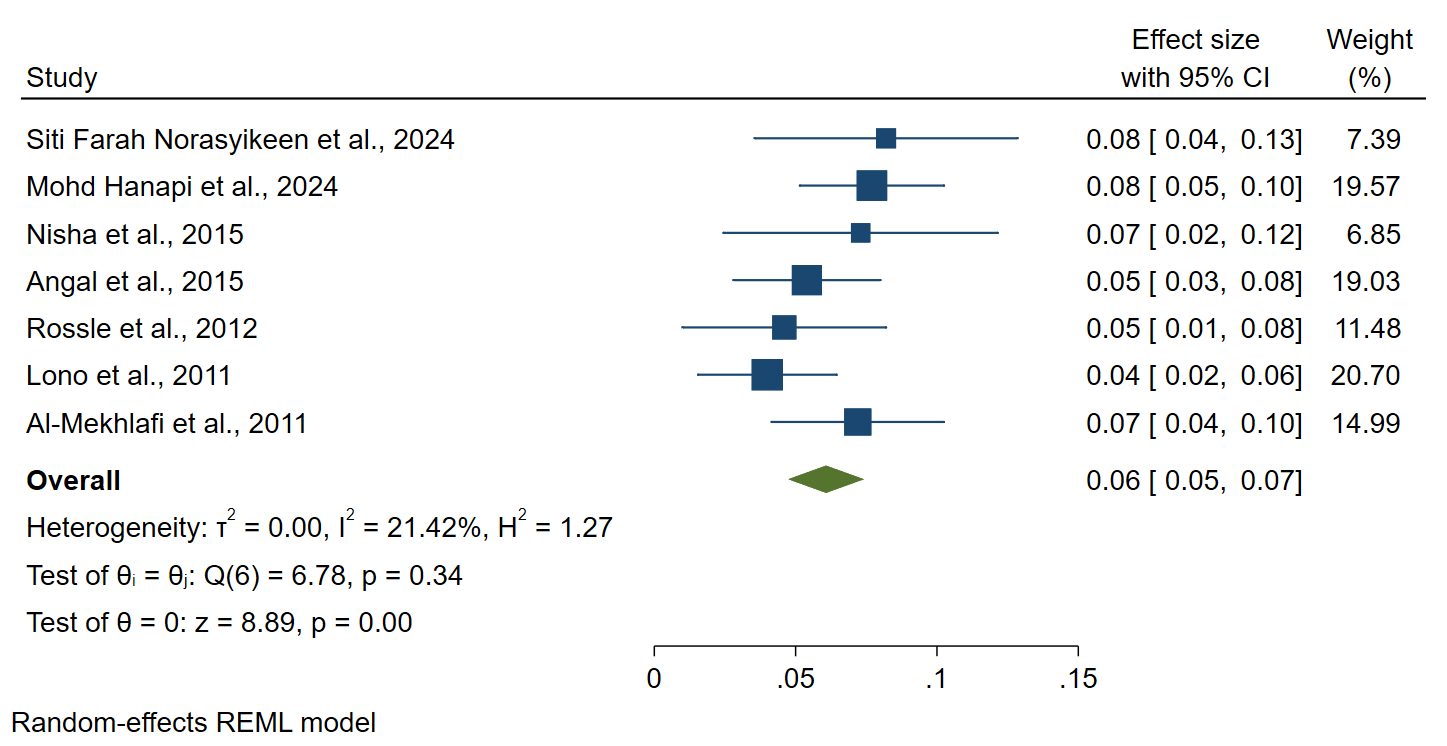


Fig S7.8 Sensitivity analysis for region in Selangor and Kuala Lumpur after removing outlier.


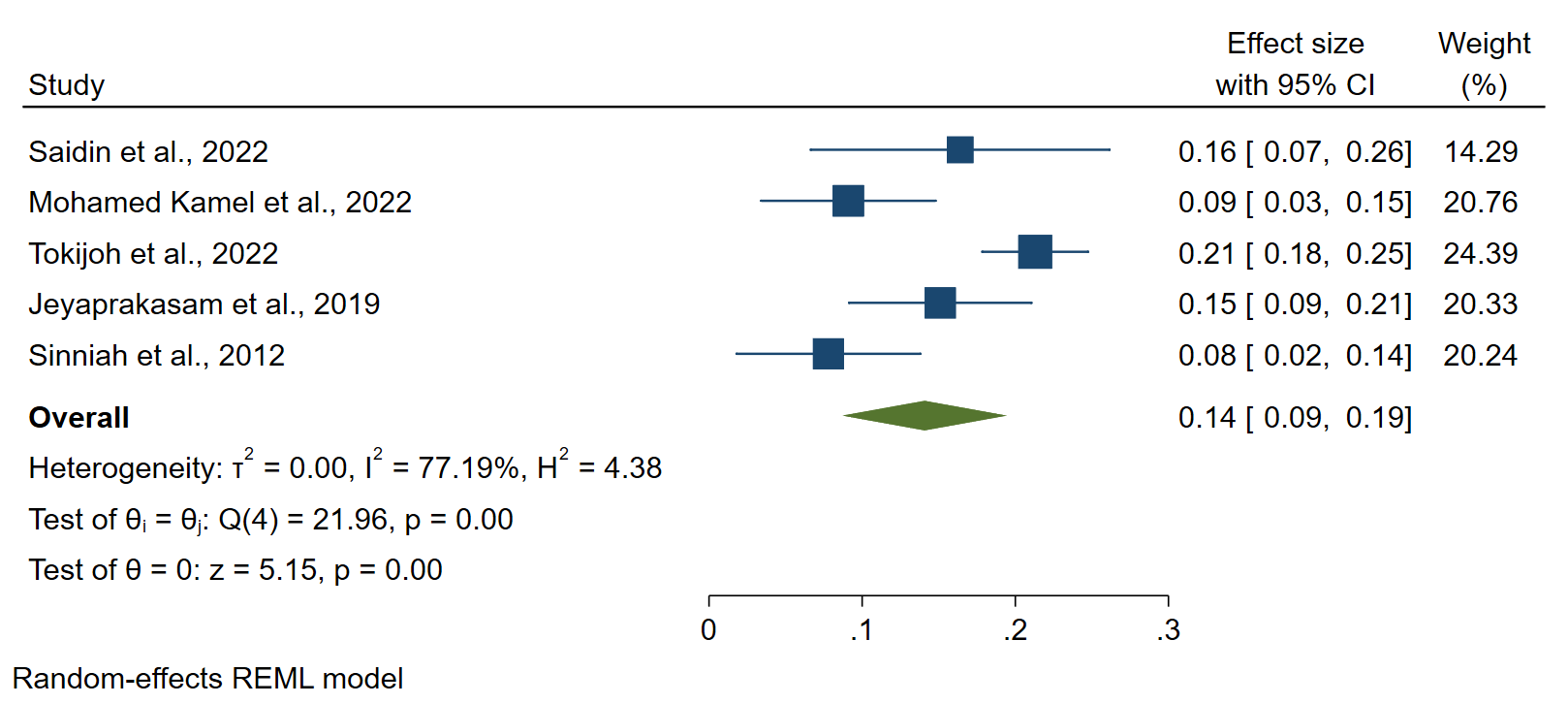


Fig S7.9 Sensitivity analysis for region in Perak after removing outlier.


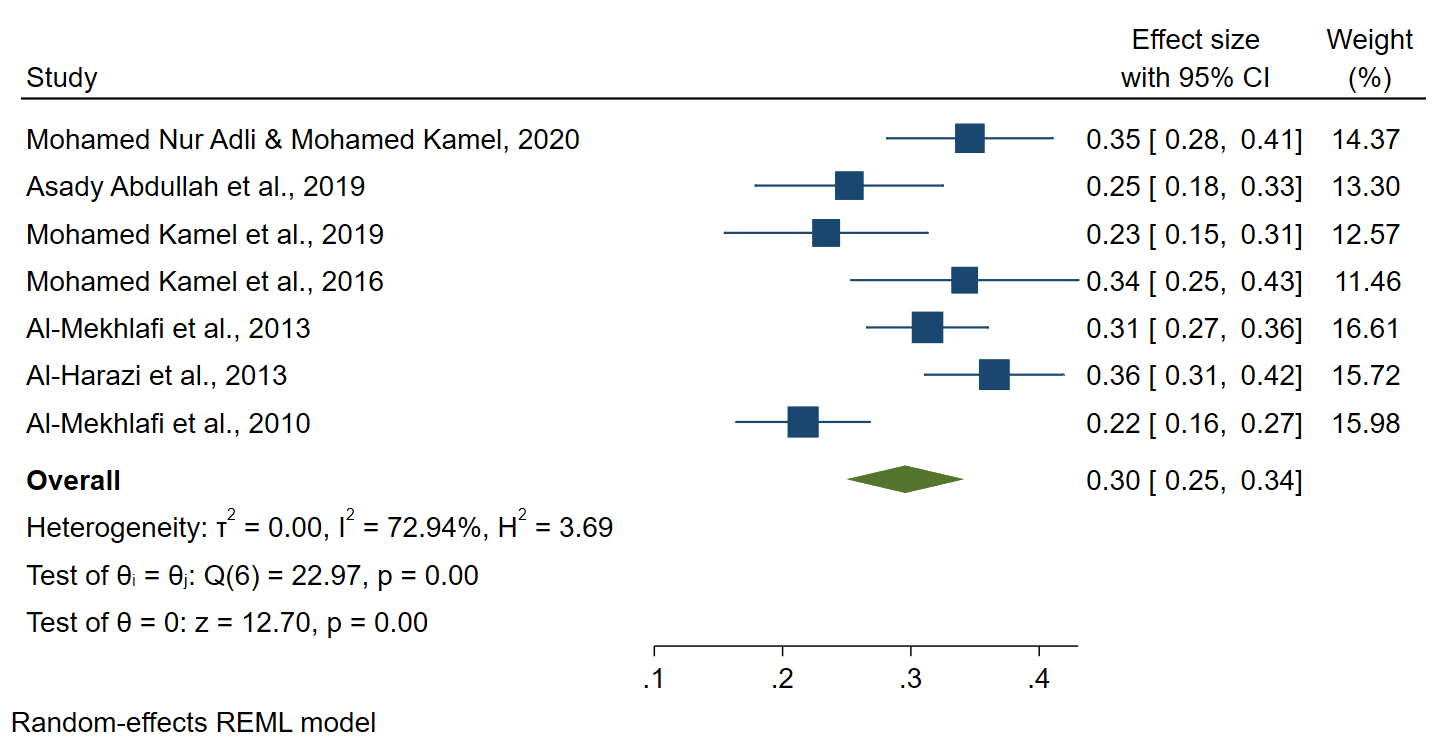


Fig S7.10 Sensitivity analysis for region in Pahang after removing outlier.


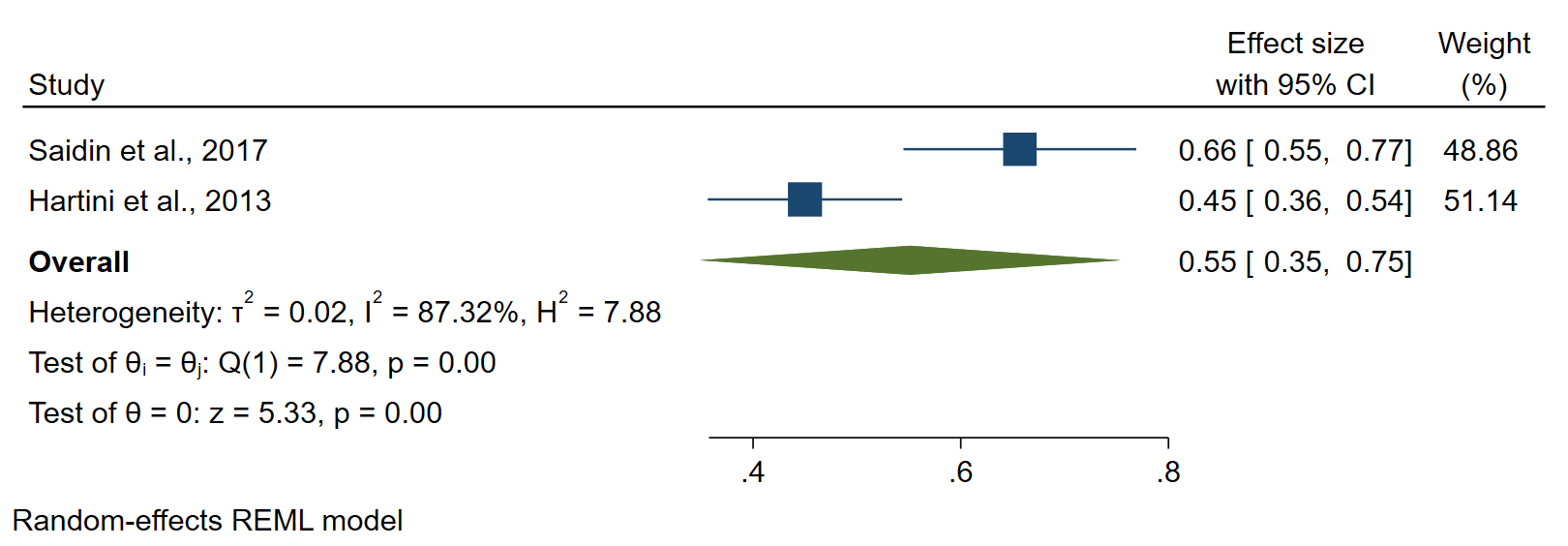


Fig S7.11 Sensitivity analysis for region in Kelantan after removing outlier.


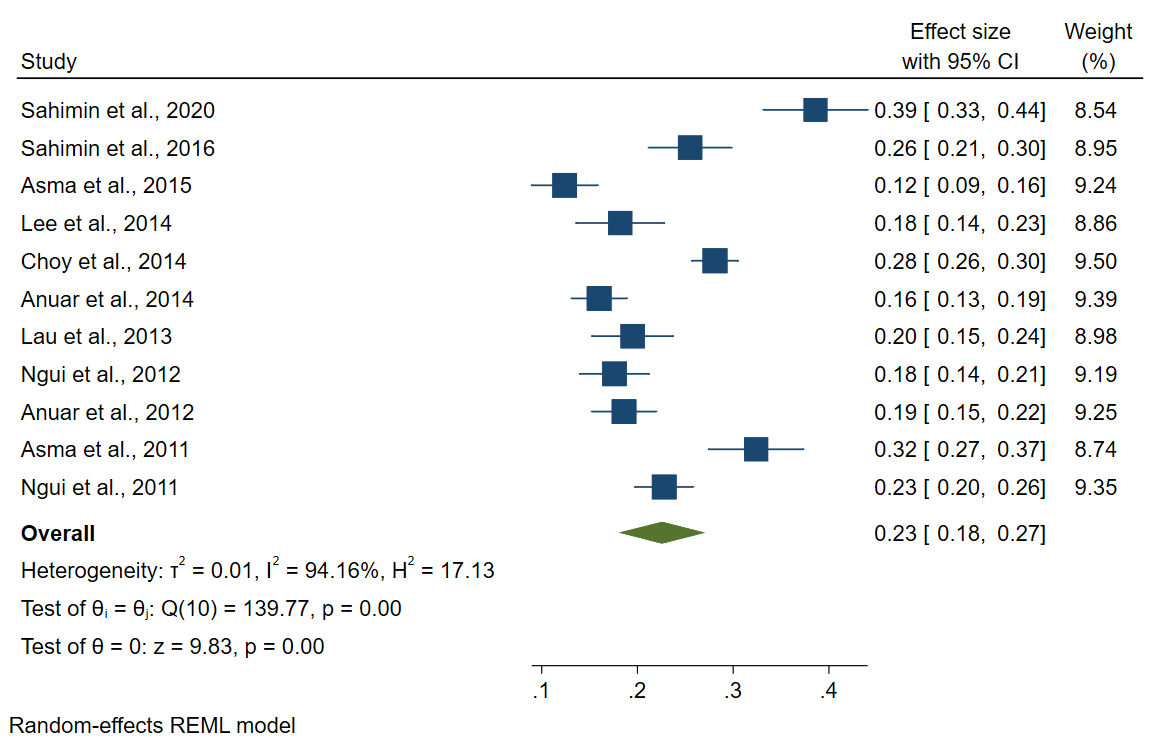


Fig S7.12 Sensitivity analysis for region in Peninsular Malaysia after removing outlier.


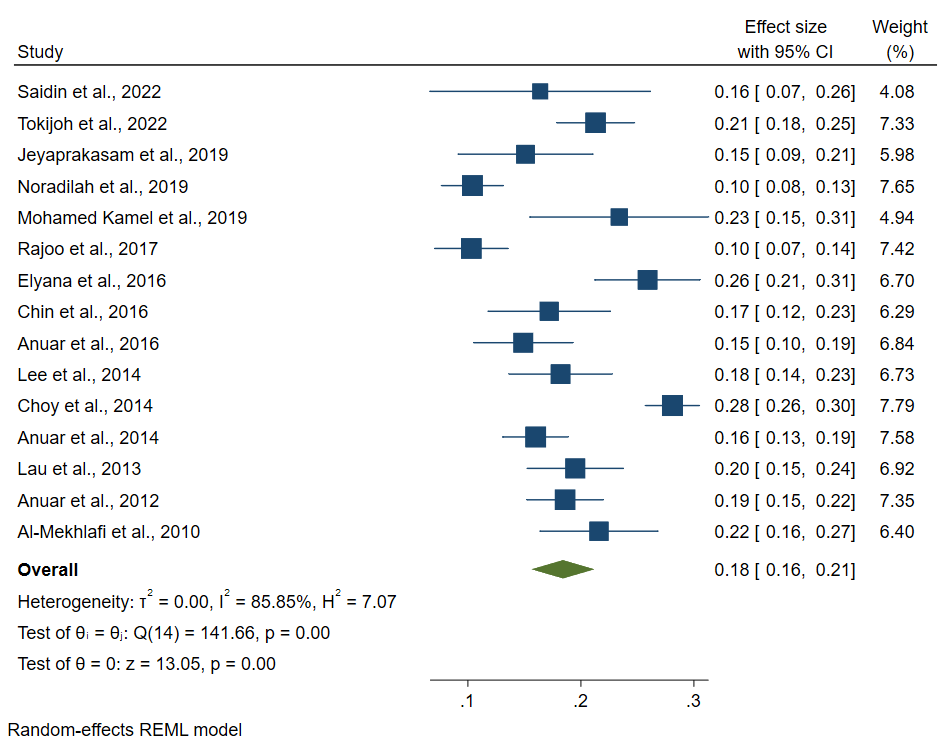


Fig S7.13 Sensitivity analysis for Aboriginal people after removing outlier.


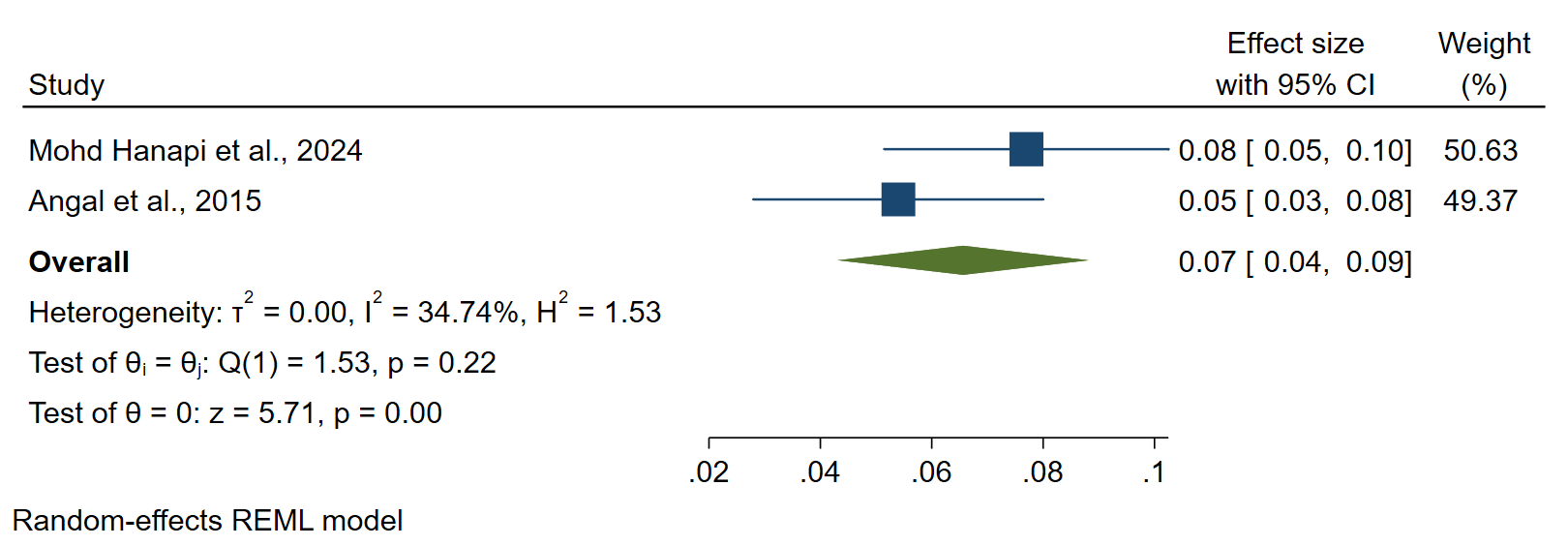


Fig S7.14 Sensitivity analysis for migrants after removing outlier.


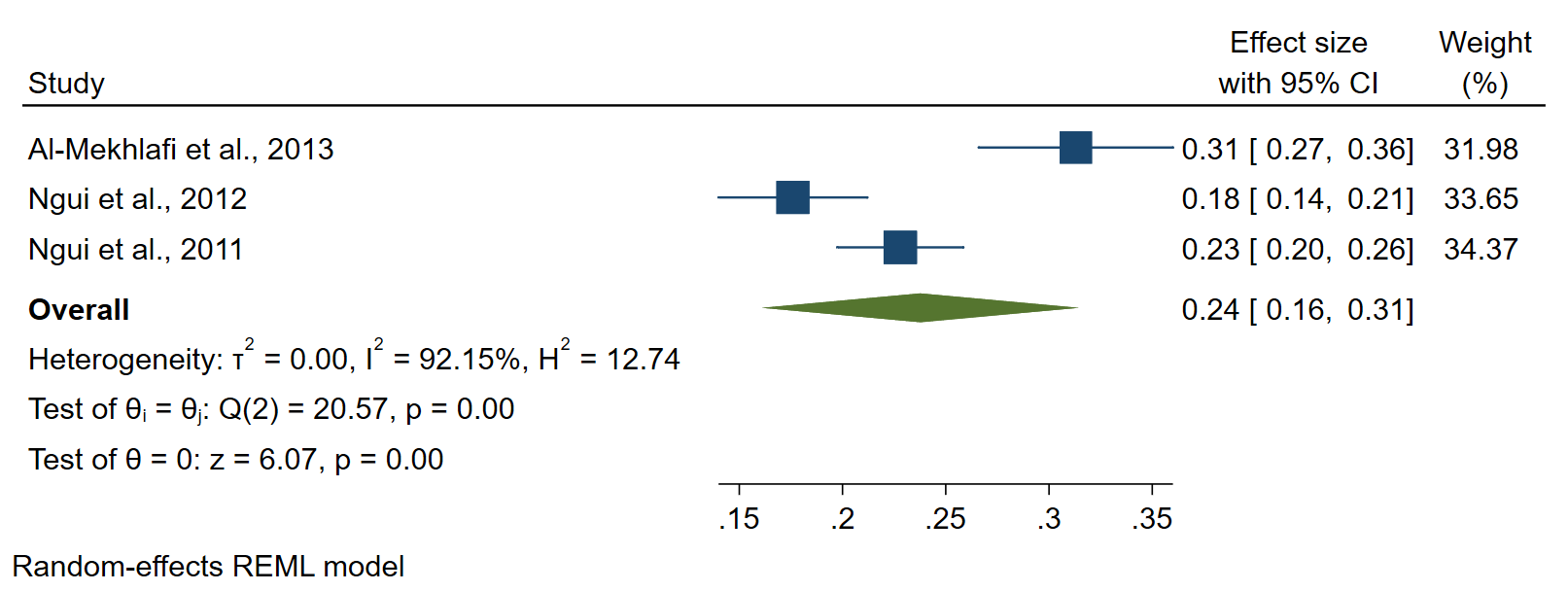


Fig S7.15 Sensitivity analysis for local communities after removing outlier.


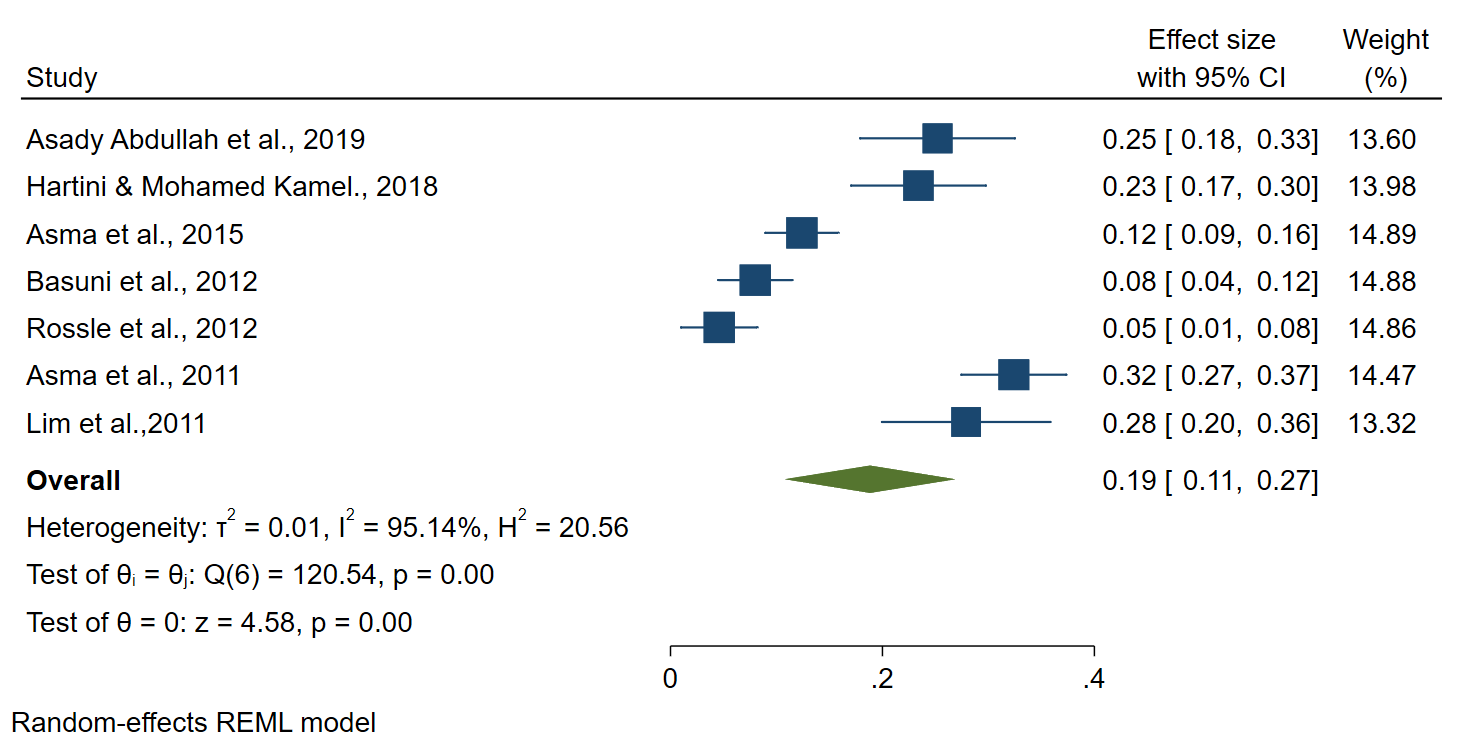


Fig S7.16 Sensitivity analysis for hospitalised patients after removing outlier.
