# Supplementary material for: Prevalence and risk factors of intestinal protozoal infections among patients in Malaysia: A systematic review and meta-analysis
Source: PLoS One. 2025 Sep 11;20(9):e0332218. doi: 10.1371/journal.pone.0332218 (PMC12425333; doi:10.1371/journal.pone.0332218)
Supplement: S8 Appendix — (DOCX) [file pone.0332218.s008.docx]

**S8 APPENDIX**

**Multivariable meta-regression model based on IPI estimates from pooled prevalence rate cases in Malaysia.**


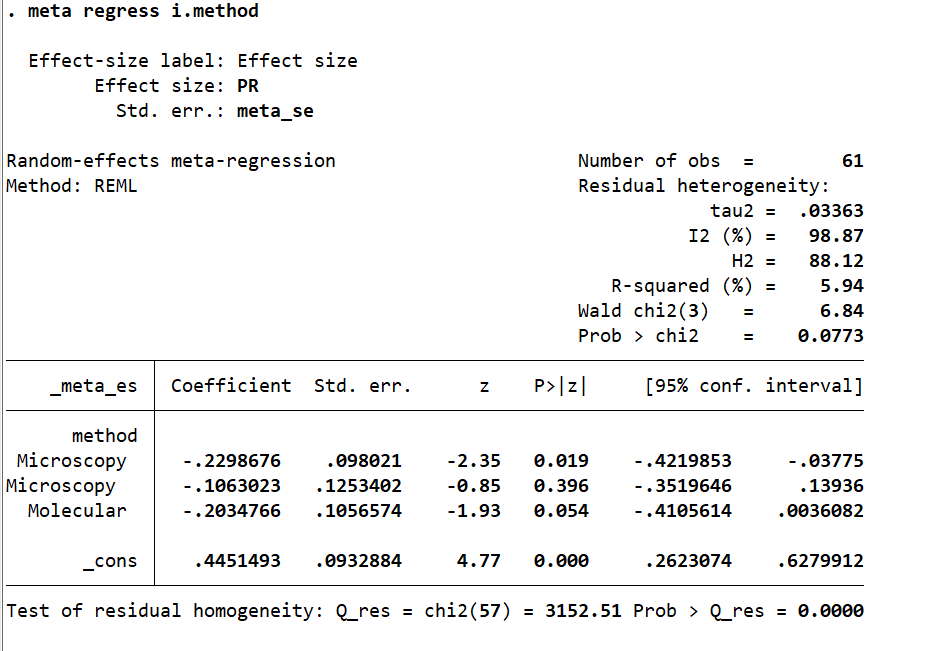


Fig 1. Meta-regression analysis for detection method.


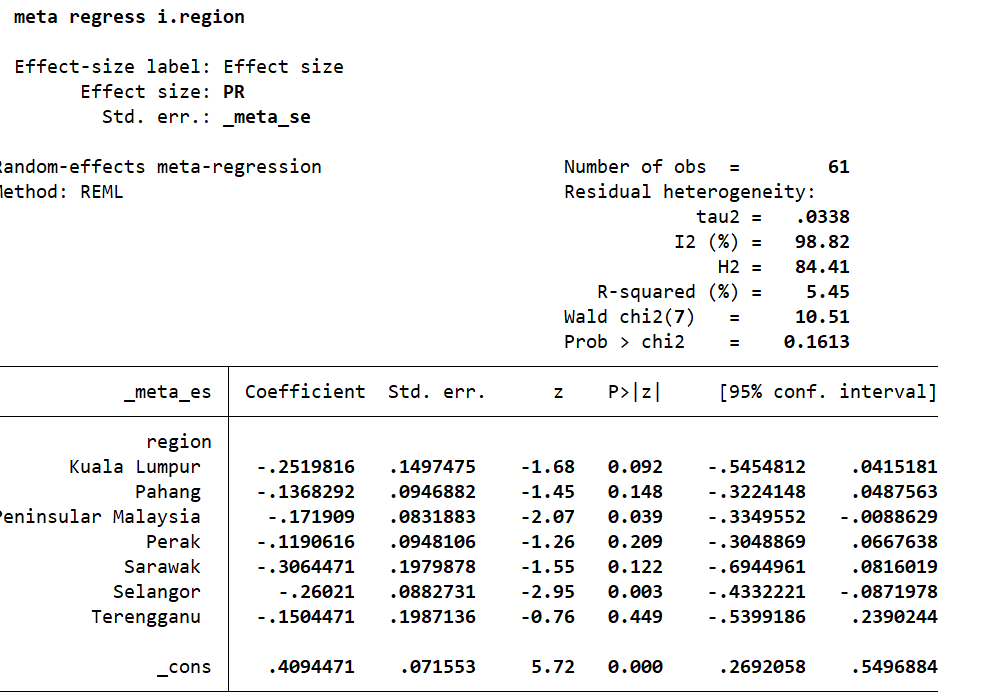


Fig 2. Meta-regression analysis for region.


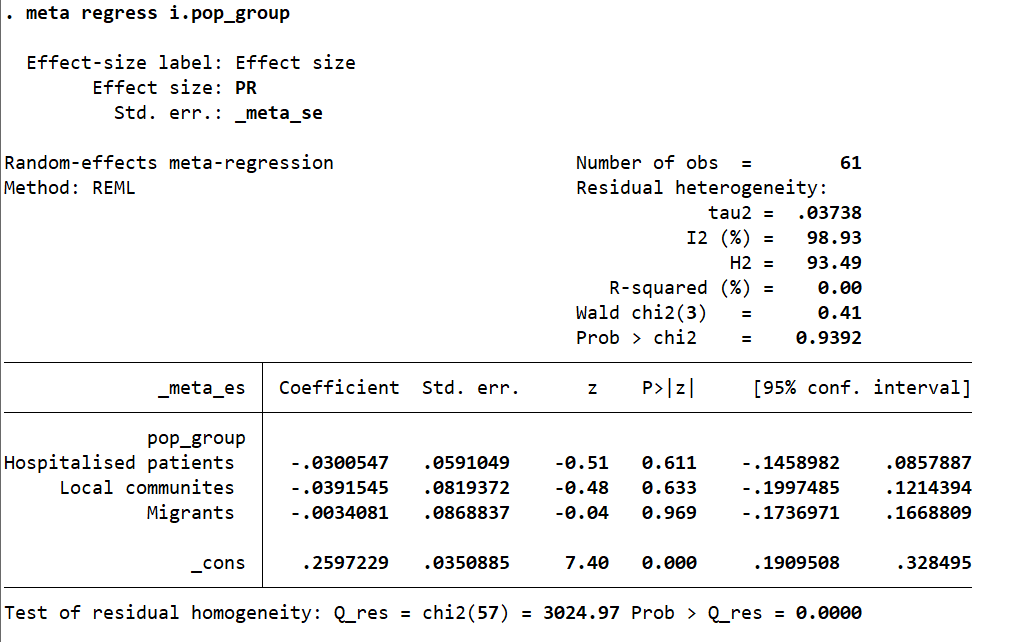


Fig 3. Meta-regression analysis for population group


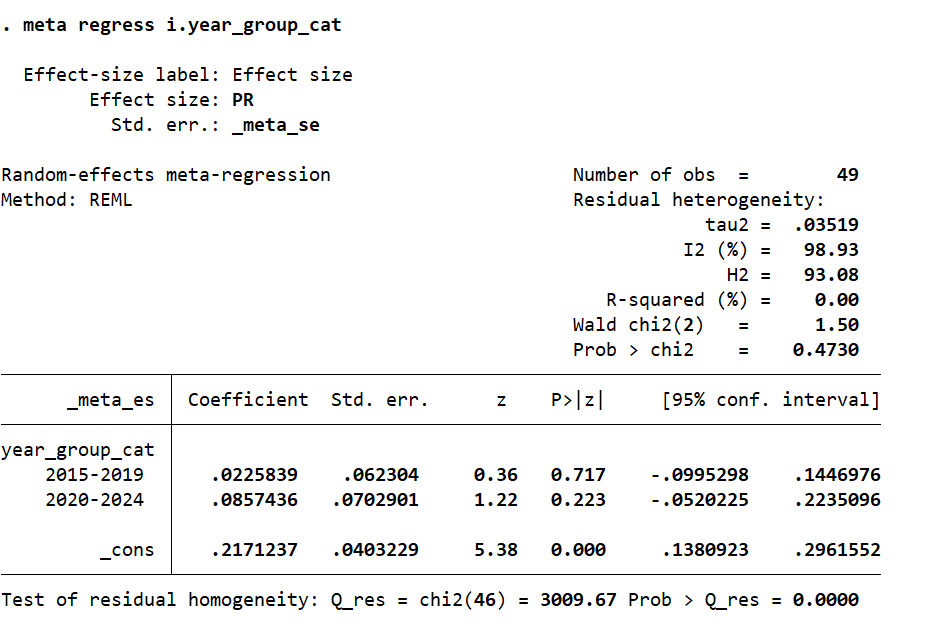


Fig 4. Meta-regression analysis for year of study


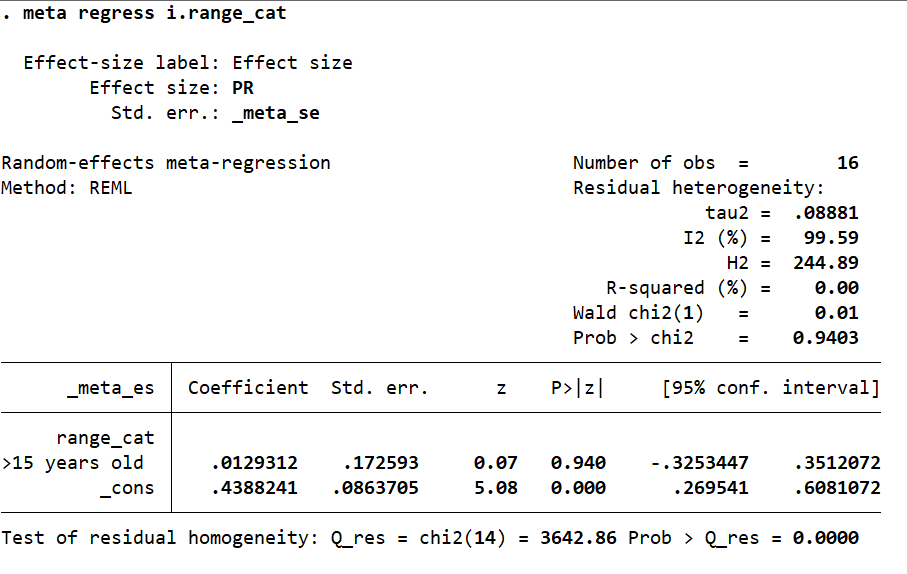


Fig 5. Meta-regression analysis for age range


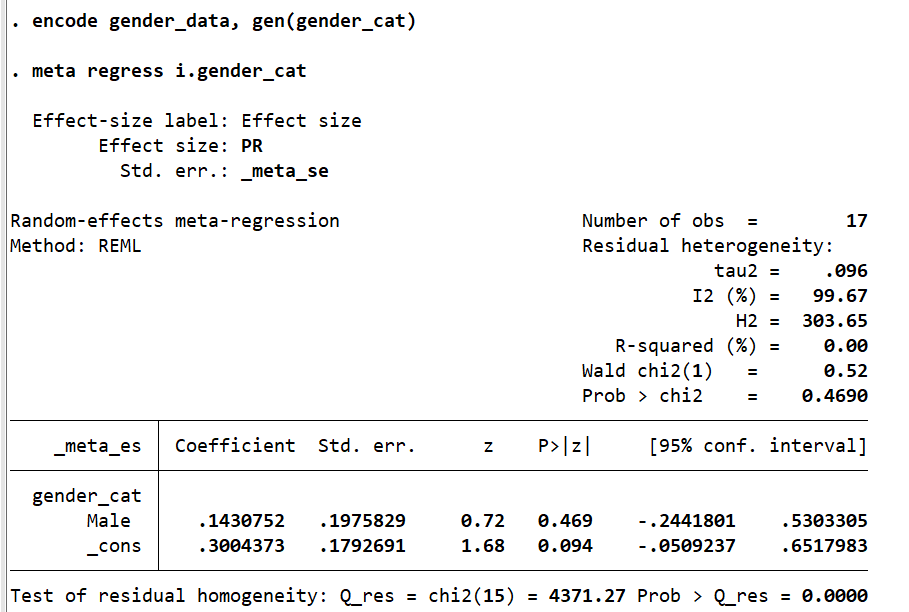


Fig 6. Meta-regression analysis for gender


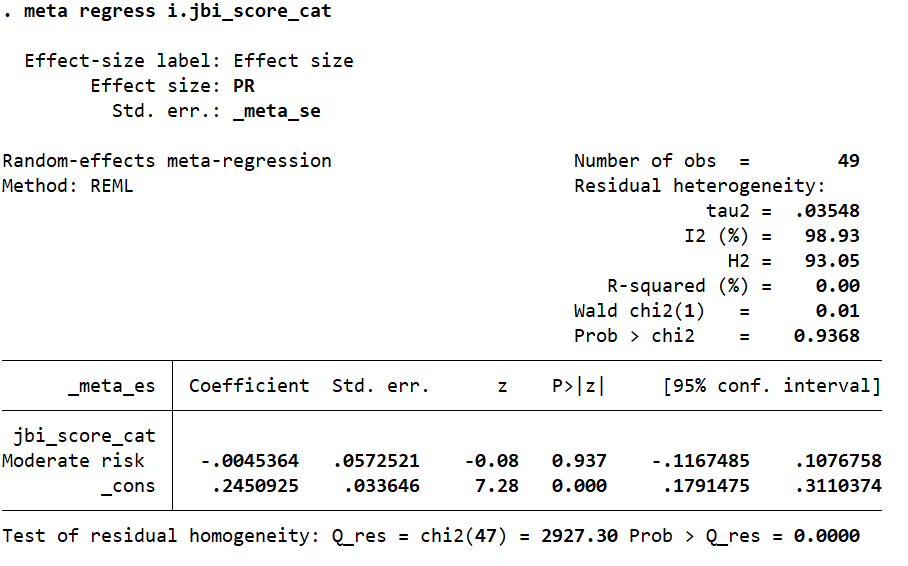


Fig 7. Meta-regression analysis for JBI score risk


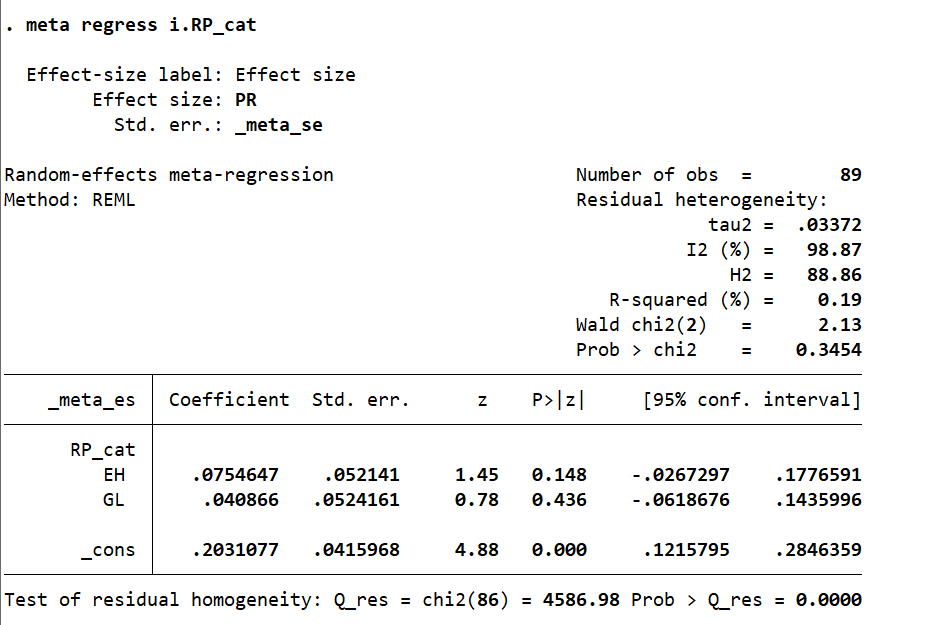


Fig 8. Meta-regression analysis for intestinal protozoa detected
