# Supplementary material for: Prevalence and risk factors of intestinal protozoal infections among patients in Malaysia: A systematic review and meta-analysis
Source: PLoS One. 2025 Sep 11;20(9):e0332218. doi: 10.1371/journal.pone.0332218 (PMC12425333; doi:10.1371/journal.pone.0332218)
Supplement: Attachment — Submitted filename: Response to Reviewers.docx [file pone.0332218.s011.docx]

**Summary of Editor Comments.**

| 1. Thank you for stating the following financial disclosure: | Response |
| --- | --- |
| Please state what role the funders took in the study.  If the funders had no role, please state: "*The funders had no role in study design, data collection and analysis, decision to publish, or preparation of the manuscript*." Please include this amended Role of Funder statement in your cover letter; we will change the online submission form on your behalf. | Thank you for your comment. We have included the following statement in the cover letter as requested:  **"The funders had no role in study design, data collection and analysis, decision to publish, or preparation of the manuscript."** |
| 2. As required by our policy on Data Availability, please ensure your manuscript or supplementary information includes the following:   1. A numbered table of all studies identified in the literature search, including those that were excluded from the analyses.  For every excluded study, the table should list the reason(s) for exclusion. | Thank you for the reminder. We have completed the required table listing all studies identified during the literature search, including those excluded from the analyses along with the reasons for exclusion. This table will be included in the supplementary files as requested **(S2** and **S3)**. |
| A table of all data extracted from the primary research sources for the systematic review and/or meta-analysis. The table must include the following information for each study:   1. Name of data extractors and date of data extraction 2. Confirmation that the study was eligible to be included in the review. 3. All data extracted from each study for the reported systematic review and/or meta-analysis that would be needed to replicate your analyses. | Thank you for the guidance. We will include the raw data extraction table in the supplementary files. The table will contain the name of data extractors and date of extraction, confirmation of study eligibility, and all data extracted from each study necessary to replicate the systematic review and/or meta-analysis. Link to the Excel file will be provided here: [meta_RAWDATA](https://docs.google.com/spreadsheets/d/1XzpjcwcpJMbELvtrSkiomoeE4N8_Sc_A/edit?usp=sharing&ouid=109442168553338519631&rtpof=true&sd=true) |
| If applicable for your analysis, a table showing the completed risk of bias and quality/certainty assessments for each study or outcome.  Please ensure this is provided for each domain or parameter assessed.   1. For example, if you used GRADE to assess certainty of evidence, provide judgements about each of the quality of evidence factor. This should be provided for each outcome. 2. An explanation of how missing data were handled 3. This information can be included in the main text, supplementary information, or relevant data repository. Please note that providing these underlying data is a requirement for publication in this journal, and if these data are not provided your manuscript might be rejected. | Thank you for your guidance.   1. We used the Joanna Briggs Institute (JBI) critical appraisal tool to assess the risk of bias for each included study. The completed risk of bias table, detailing assessments across each domain, will be provided in the supplementary files (**S6**) 2. Information regarding the handling of missing data is described in the **Data Extraction** section on *Page 6* of the manuscript (highlighted in yellow),   *“For studies with missing or incomplete data…. the quantitative analysis.”*   3. We have included the risk of bias assessments and approach to missing data, presented in both the main text and supplementary materials |

| Comment No | Comment | Response |
| --- | --- | --- |
| Reviewer 1(Highlighted in yellow) | | |
| 1 | What methodological approach was used to assess the overall quality of the studies, and how did it categorize the studies based on their scores? | Thank you for the valuable comment. We have mentioned the methodological approach on *Quality assessment* section on page 6 and 7, highlighted line  “Risk *of bias (quality assessment) was assessed using the Joanna Briggs Institute critical appraisal tools [20] …* *observational study designs.”* |
| 2 | How did the different diagnostic methods (microscopy, molecular, immunoassay, and combinations thereof) impact the reported prevalence rates of intestinal protozoal infections? | Thank you for the valuable comment. We have mentioned it discussion section on page 21, highlighted line*.*  *“*This *trend is reflected in our findings where ……. intestinal protozoal infections.”* |
| 3 | What does the high heterogeneity index (I²) reported in the meta-analysis suggest about the included studies, and how might this affect the interpretation of the pooled prevalence rates? | Thank you for the valuable comment. We have mentioned it data synthesis section on page 7 and 8, highlighted line*.*  *” The heterogeneity index (I²) indicates considerable variability …. proportions and 95% confidence intervals.”* |
| 4 | According to the article, how did the prevalence of intestinal protozoal infections differ across geographical regions such as Perak, Selangor, and Pahang, and what might explain these variations? | Thank you for the valuable comment. We have mentioned it discussion section on page 22, highlighted line*.*  *”* *The different prevalence in the individual regions can be explained by differences in ……observed regional differences”* |
| 5 | Discuss the significance of the Egger’s test result in evaluating the presence of publication bias. How does this result align with the visual interpretation of the funnel plot? | Thank you for the valuable comment. We have mentioned it discussion section on page 13, highlighted line  *“To confirm this statistically, the Egger test’s method…...robustness of the overall results”* |
| 6 | What socioeconomic and hygiene-related risk factors were most strongly associated with increased intestinal protozoal infections, and how did the authors support these conclusions? | Thank you for the valuable comment. We have mentioned it discussion section on page 24, highlighted line  *“These conclusions arise from consistent patterns observed in the included studies…...infections in Malaysia.”* |
| 7 | In the article, infectious agents such as *Entamoeba spp*., *Giardia lamblia*, and *Cryptosporidium spp*. are mentioned. How does the prevalence of these species compare, and how should they be formatted when writing about them? | Thank you for your comment. We have clarified the comparison of species-specific prevalence in the discussion section on page 23, highlighted in yellow.  *“In Malaysia, Entamoeba spp. had the highest prevalence of intestinal protozoal infections (18%), followed by Giardia lamblia (11%) and Cryptosporidium spp. (9%).”*  *.*  We also confirm that all species names have been formatted according to scientific conventions. In line with taxonomic standards, genus and species names (e.g., *Entamoeba spp.*, *Giardia lamblia*, *Cryptosporidium spp.*) are italicised, with the genus capitalised and the species in lowercase. This formatting follows the standard conventions for scientific names of microorganisms, where genus and species names are italicized, the genus name is capitalized, and species names or abbreviations such as "*sp*." (single unnamed species) or "*spp.*" (multiple unnamed species) are lowercase and italicized |
| 8 | How does the sensitivity analysis strengthen the reliability of the meta-analysis findings, particularly in light of the identified publication bias? | Thank you for the valuable comment. We have mentioned it discussion section on page 26, highlighted line  *“To assess the impact of this bias, a sensitivity…. the meta-analysis conclusions.”* |
| Reviewer 2 (highlighted in GREEN) | | |
| 1 | High heterogeneity (I² = 98.22%): The extreme heterogeneity undermines the reliability of pooled estimates. The authors should perform meta-regression to identify sources of variability (e.g., regional differences, diagnostic methods, population characteristics). | Thank you for your comment. We have conducted the meta-regression analysis as requested, and the results are presented on *Page 20* of the manuscript.  Meta regression analysis has been added in supplementary file in S8. |
| 2 | Egger’s test confirms significant publication bias. A sensitivity analysis (e.g., the trim-and-fill method) should be conducted to assess the impact of missing studies. | Thank you for your comment. We have conducted the sensitivity analysis as requested, and the results are presented on *Page 19* of the manuscript. |
| 3 | Missing supplementary materials: Figures (e.g., PRISMA flowchart, forest plots) and supplementary tables are referenced but not provided. These must be included. | Thank you for your observation. We have acknowledged the omission and ensure that all referenced supplementary materials, including the PRISMA flowchart, forest plots, and supplementary tables, are provided in the revised submission. |
| 4 | Diagnostic limitations: Overreliance on microscopy (28/51 studies) likely underestimates species-specific prevalence (e.g., inability to distinguish *E. histolytica* from *E. dispar*). This limitation should be explicitly discussed | Thank you for the valuable comment. We have adjusted the data, and the necessary amendments have been made on *Page 21*, highlighted in green.  *“The over-reliance on microscopy in more…. prevalence estimates.”* |
| 5 | Regional disparities: Peninsular Malaysia is over-represented (18 studies) vs. East Malaysia (1 study). The authors should acknowledge and discuss the implications. | Thank you for the valuable comment. We have mentioned it discussion section on page 22, highlighted line  *“However, it is important to acknowledge…to better inform nationwide public health strategies.*  *.”* |
| 6 | Language bias: Excluding non-English studies may omit locally relevant data. Future reviews should incorporate multilingual databases to mitigate this. | Thank you for your comment. We acknowledge the concern regarding language bias. However, we would like to clarify that non-English studies were not excluded from this review. Articles published in both English and Bahasa Malaysia were included during the screening process to ensure broader representation of locally relevant data.  . |
| 7 | Discussion enhancements: Expand on how diagnostic method variability affects prevalence estimates. Discuss strategies to improve IPI surveillance (e.g., standardized molecular diagnostics). | Thank you for the valuable comment.    We have mentioned the diagnostic variability in discussion section on page 21, highlighted line*.*  *“*This *trend is reflected in our findings where……. intestinal protozoal infections.”*  Next, we also have discussed the strategies in discussion section on page 22 and 23, highlighted line  *“To improve IPI surveillance, it is essential to implement…….and comparable reporting across studies.”* |
| 8 | Limitations section: Explicitly state how high heterogeneity, regional disparities, and diagnostic limitations impact conclusions. | Thank you for the valuable comment. We have mentioned it discussion section on page 27, highlighted line  “*This review has several limitations. …. diagnostic tools in future studies.”* |
| 9 | The manuscript is largely an extension of your previously published protocol. Please clarify how this study builds upon it. | Thank you for the valuable comment. We have mentioned it introduction section on page 5, highlighted line  *“This study builds on our previously …... in the Malaysian context [87].”* |
| Reviewer 3- Highlighted in BLUE | | |
| 1 | The title suggests a focus on both prevalence and risk factors, but the latter is addressed only descriptively. Consider a formal meta-analysis of risk estimates (e.g., OR, RR) or revise the title. | Thank you for your insightful comment. We have taken your suggestion into account and made amendments to include a more formal meta-analysis of risk factor estimates, rather than addressing them descriptively. These updates are reflected in the revised manuscript. |
| 2 | Include the full search strategy used for each database in the supplementary materials. | Thank you for your suggestion. We will include the full search strategy for each database in the supplementary materials (**S1**) in the revised submission. |
| 3 | Present a summary of JBI scores and classify the quality of studies (high/moderate/low). | Thank you for your helpful suggestion. We have included a summary of the JBI scores and the classification of study quality (high/moderate/low) in **Table 3** of the manuscript. The full assessment details can also be found in the **supplementary materials (S6)** |
| 4 | Explore heterogeneity more robustly with expanded meta-regression and sensitivity analysis. | Thank you for your comment. We have conducted the meta-regression and sensitivity analysis as requested, and the results are presented on *Page 19 and 20* of the manuscript |
| 5 | Correct for publication bias using appropriate methods (e.g., Trim and Fill). | Thank you for your comment. We have conducted the sensitivity analysis as requested, and the results are presented on *Page 19* of the manuscript. |
| 6 | Address minor language and structural inconsistencies. | Thank you for the feedback. We have carefully reviewed the manuscript to address minor language and structural inconsistencies and have made the necessary revisions to improve clarity and readability. |
