# Supplementary material for: Prevalence and risk factors of intestinal protozoal infections among patients in Malaysia: A systematic review and meta-analysis
Source: PLoS One. 2025 Sep 11;20(9):e0332218. doi: 10.1371/journal.pone.0332218 (PMC12425333; doi:10.1371/journal.pone.0332218)
Supplement: Attachment — Submitted filename: Response_to_Reviewers_auresp_2.docx [file pone.0332218.s012.docx]

Response to Reviewer

| **Section** | **Reviewer Comment** | **Response** |
| --- | --- | --- |
| **Abstract** | The abstract is concise and summarizes the key findings effectively. However, consider adding a sentence about the clinical or public health implications of the 24% pooled prevalence to highlight the study's significance. | Thank you for the suggestion. We have revised the abstract to include a sentence highlighting the clinical and public health implications of the 24% pooled prevalence, emphasizing its significance for disease control and prevention strategies highlighting in yellow in page 3  *“The high pooled prevalence of 24% underscores a ……. reducing prevalence and transmission.”* |
| **Introduction** | Please consider briefly mentioning the global burden of IPI to contextualize the Malaysian data. | Thank you for the suggestion. We have revised the introduction to include a sentence highlighting the global burden of IPI to contextualize the Malaysian data highlighting in yellow in page 4.  *“Given that IPI remain a significant …. local share of this worldwide burden. [2].* |
| **Methods** | Clarify why studies from East Malaysia were excluded due to a focus on animal/environmental samples. This could introduce bias, and a brief justification would strengthen the manuscript. | Thank you for your comment. We have added a justification in the Methods section to clarify that studies from East Malaysia were excluded because they primarily focused on animal or environmental samples, which were outside the scope of this review that targeted human infection prevalence. We acknowledge that this exclusion could introduce bias and have mentioned it as a limitation. We have highlighted the amendment in yellow in page 6.  *“Most East Malaysian studies …… fell outside our scope.*  In response to this comment, one additional reference has been included, and the reference list has been updated to reflect this change highlighted in green page 37. |
| **Results** | Consider adding a brief summary of the risk factors in the main text to complement Table 5. | Thank you for your comment. We have added brief summary of the risk factors in the main text to complement Table 5. We have highlighted the amendment in yellow in page 11.  *“A subgroup meta-analysis revealed a higher prevalence among ……eating raw vegetables (40%).”* |
|  | Again, the statistical methods are appropriate, but the high heterogeneity (I² > 98%) should be discussed earlier in the results section to prepare readers for its implications. | Thank you for the suggestion. We have revised the Results section to mention the high heterogeneity (I² > 98%) earlier, immediately after reporting the pooled prevalence, to provide readers with context and prepare them for its implications. We also added a brief explanation regarding potential sources of heterogeneity. The amendment was highlighted in yellow in page 9.  *“A high level of heterogeneity…... diagnostic methods and study designs.”* |
| **Discussion** | Please strengthen the section on limitations by explicitly stating how the high heterogeneity and regional disparities might affect the generalizability of the findings. | Thank you for the suggestion. We have revised the Limitations section to explain how high heterogeneity and regional disparities may affect the generalizability of the pooled prevalence estimates. The amendment was highlighted in yellow in page 27.  *“In addition, most of …… to the entire Malaysian population.”* |
|  | The public health recommendations (e.g., improved water access, hygiene education) are relevant but could be expanded with specific policy suggestions. | Thank you for the suggestion. We have expanded the recommendation on public health with specific policy suggestions. The amendment was highlighted in yellow in page 27.  *“Specific policy measures could be implemented such as …… infections on public health could be steadily reduced.”* |
| **Tables & Figures** | Ensure that the supplementary files (e.g., PRISMA flowchart, forest plots) are referenced in the main text where appropriate. | Thank you for the suggestion. We have referred the supplementary files in the main text where appropriate. The amendment was highlighted in green.  S1– Page 5  S2 – Page 6  S3 – Page 6  S4 – Page 10  S5 – Page 7  S6 – Page 8  S7 – Page 12  S8 – Page 12  S9 – Page 5 |
| **References** | Check for consistency in formatting (e.g., italics for species names, journal abbreviations). | Thank you for highlighting this. We have carefully reviewed the manuscript to ensure consistent formatting, including italicizing all species names and standardizing journal abbreviations according to the journal’s style guidelines. |
