# Supplementary material for: Prevalence and risk factors of intestinal protozoal infections among patients in Malaysia: A systematic review and meta-analysis
Source: PLoS One. 2025 Sep 11;20(9):e0332218. doi: 10.1371/journal.pone.0332218 (PMC12425333; doi:10.1371/journal.pone.0332218)
Supplement: Attachment — Submitted filename: Response_to_Reviewers_auresp_3.docx [file pone.0332218.s013.docx]

**Response to Reviewer**

| **Section** | **Reviewer Comment** | **Response** |
| --- | --- | --- |
| **Abstract** | The abstract is concise and informative but could briefly mention the high heterogeneity observed (I² > 98%) and its implications for interpreting the pooled prevalence estimates. | Thank you for this valuable suggestion. We have revised the abstract to briefly mention the high heterogeneity (I² > 98%) and highlighted its implications for interpreting the pooled prevalence estimates highlighted in green on page 3.  *Page 3 “A high level…the included studies.”*  And  *The pooled prevalence.… generalizable to all settings*. |
| **Introduction** | The background effectively contextualizes the study, but the transition to the Malaysian context could be smoother. Consider adding a sentence or two about why Malaysia is a critical region for studying IPI (e.g., diverse populations, urbanization vs. rural disparities, etc.). | Thank you for your suggestion. We have revised the background to include a clearer transition to the Malaysian context by highlighting why Malaysia is an important setting for studying intestinal protozoal infections, considering its diverse populations and rural–urban disparities highlighted in green on page 4.  *“Given that IPI remain a significant global health concern, Malaysia is a valuable setting for…...in access to healthcare, water quality, and sanitation.”* |
| **Methods** | **Search Strategy:** The inclusion of multiple databases is commendable. However, the exclusion of East Malaysian studies due to their focus on animal/environmental samples should be explicitly justified in the main text (not just in the response to reviewers) to avoid potential bias. | Thank you for this helpful suggestion. We have revised the manuscript to explicitly state the rationale for excluding East Malaysian studies from animal and environmental studies. This clarification has been added to the main text in *Methods* section on page 6, highlighted in green.  *“The limited availability …. human infections, or did not examine the protozoa of interest.”* |
|  | **Quality Assessment:** The Joanna Briggs Institute tool is appropriate, but the results of the quality assessment (e.g., high risk of bias in some studies) should be discussed in the limitations section. | Thank you for your comment. In our quality assessment using the Joanna Briggs Institute tool, none of the included studies were rated as high risk. However, several were rated as moderate quality. We have revised the *Limitations* section to indicate that the inclusion of studies with moderate methodological quality may have influenced the pooled prevalence estimates and contributed to the observed heterogeneity. This clarification has been added to the manuscript on page 28, highlighted in yellow.  *“Although none of the included ……… observed heterogeneity.”* |
| **Results** | **Subgroup Analyses:** The regional and population-based subgroup analyses are well-presented. However, the high heterogeneity (I² > 98%) suggests significant variability across studies. The authors should discuss potential reasons (e.g., differences in diagnostic methods, study populations) and how this affects the interpretation of pooled estimates. | Thank you for the valuable suggestion. We have revised the discussion to explicitly address the high heterogeneity (I² > 98%) observed in the subgroup analyses and to briefly note potential sources of variability. The revised text has been incorporated on page 24 and highlighted in green  *“This heterogeneity likely …., it should be interpreted cautiously in light of these variations.”* |
|  | **Risk Factors:** The meta-analysis of risk factors is a strength, but the results should be interpreted cautiously due to residual confounding. Consider adding a sentence about this limitation. | Thank you for this helpful suggestion. We have revised the *Result* section. This clarification has been added on page 12, highlighted in green.  *‘The results should be interpreted with caution as…. influences may still have affected the results.”* |
| **Discussion** | **Clinical and Public Health Implications:** The discussion effectively highlights the need for interventions (e.g., improved sanitation, hygiene education). However, it could be strengthened by proposing specific, actionable recommendations for policymakers (e.g., targeted screening in high-risk groups like indigenous communities). | Thank you for this constructive suggestion. We have revised the *Discussion* to include specific, actionable recommendations for policymakers. This paragraph has been included on page 26, highlighted in green.  *“In terms of clinical and public health practise, our findings emphasise the need for measures that go beyond general……. for both clinical management and public health.”* |
| **Limitation** | The limitations section is thorough but should explicitly mention the potential for publication bias (as indicated by the funnel plot and Egger test) and its impact on the results. | Thank you for your comment. We have revised the *Limitations* section. This clarification has been added to the manuscript on page 28, highlighted in green.  *“Although none of the included studies were categorised as high risk of bias using the Joanna Briggs Institute tool, some of them…. observed heterogeneity.”* |
| **Tables & Figures** | Table 3: Ensure all abbreviations (e.g., "Ref") are defined in the footnote. | Thank you for your comment. We would like to clarify that the abbreviation “Ref” only appears in **Table 7**, not Table 3. We have revised Table 7 accordingly to ensure that “Ref” and all other abbreviations are clearly defined in the footnote. |
|  | Figure 6 (Funnel Plot): The asymmetry suggests possible publication bias. This should be discussed in the limitations. | Thank you for highlighting this. The observed asymmetry in Figure 6 has been acknowledged, and a note on potential publication bias has now been incorporated into the L*imitations* section on page 28, highlighted in green.  *“Possible publication bias was analysed using the funnel …...cannot be completely ruled out and should be considered when interpreting the results.”* |
| **References** | The references are comprehensive and up-to-date. However, check for consistency in formatting (e.g., italicization of species names, journal abbreviations). | Thank you for highlighting this. We have carefully reviewed the manuscript to ensure consistent formatting, including italicizing all species names and standardizing journal abbreviations according to the journal’s style guidelines. |
| **Supplementary materials** | Ensure all supplementary files (e.g., PRISMA flowchart, forest plots) are referenced in the main text and uploaded correctly. | Thank you for your comment. We have ensured that all supplementary files, including the PRISMA flowchart and forest plots, are properly referenced in the main text and uploaded correctly. |
